# *Agathis robusta* Bark Extract Protects from Renal Ischemia-Reperfusion Injury: Phytochemical, *In Silico* and *In Vivo* Studies

**DOI:** 10.3390/ph15101270

**Published:** 2022-10-14

**Authors:** Maged E. Mohamed, Nora Tawfeek, Samar S. Elbaramawi, Mahmoud H. Elbatreek, Eman Fikry

**Affiliations:** 1 Department of Pharmaceutical Sciences, College of Clinical Pharmacy, King Faisal University, Al-Ahsa 31982, Saudi Arabia; 2 Department of Pharmacognosy, Faculty of Pharmacy, Zagazig University, Zagazig 44519, Egypt; 3 Department of Medicinal Chemistry, Faculty of Pharmacy, Zagazig University, Zagazig 44519, Egypt; 4 Department of Pharmacology and Toxicology, Faculty of Pharmacy, Zagazig University, Zagazig 44519, Egypt

**Keywords:** *Agathis* *robusta*, renal ischemia-reperfusion, acute kidney injury, network pharmacology

## Abstract

Background: Acute kidney injury (AKI) induced by renal ischemia-reperfusion injury (RIRI) is associated with a high incidence of mortality. Existing therapies are mainly supportive, with no available nephroprotective agent. The purpose of this study is to examine the potential protective effect of *Agathis robusta* Bark Extract (ARBE) in RIRI. Methods: The chemical composition of ARBE was examined by LC-ESI-MS/MS. Network pharmacology was utilized to identify the RIRI molecular targets that could be aimed at by the identified major components of ARBE. Experimentally validated protein–protein interactions (PPIs) and compound-target networks were constructed using the STRING database and Cytoscape software. Molecular docking studies were employed to assess the interaction of the most relevant ARBE compounds with the hub RIRI-related targets. Furthermore, ARBE was tested in a rat model of RIRI. Results: The phytochemical analysis identified 95 components in ARBE, 37 of which were majors. Network analysis identified 312 molecular targets of RIRI that were associated with ARBE major compounds. Of these 312, the top targets in the experimentally validated PPI network were HSP90, EGFR, and P53. The most relevant compounds based on their peak area and network degree value included narcissoside, isorhamnetin-3-*O*-glucoside, and syringetin-3-*O*-glucoside, among others. Docking studies of the most relevant compounds revealed significant interactions with the top RIRI-related targets. In the in vivo RIRI experiments, pretreatment of ARBE improved kidney function and structural changes. ARBE reduced the renal expression of p-NfkB and cleaved caspase-3 by downregulating HSP90 and P53 in rats exposed to RIRI. Conclusion: Taken together, this study revealed the chemical composition of ARBE, depicted the interrelationship of the bioactive ingredients of ARBE with the RIRI-related molecular targets, and validated a nephroprotective effect of ARBE in RIRI.

## 1. Introduction

Acute kidney injury (AKI) characterized by abrupt decline in renal function, is associated with several short and long-term complications and a high risk for mortality [1]. Renal ischemia-reperfusion injury (RIRI) is a common cause of AKI that occurs in clinical settings, e.g., kidney transplantation, heart surgery, and shock [2]. Management of this condition is mainly supportive, and survivors have markedly reduced health-related quality of life (HRQL) and continue to develop long-term adverse outcomes, including chronic kidney disease (CKD) [3]. Therefore, continuous search for novel nephroprotective agents, including natural products, in the setting of RIRI-induced AKI is essential.

Conifers constitute the main and most distinct set of living gymnosperms and include more than 600 species and 60–65 genera distributed in seven families, the Pinaceae, Cupressaceae, Podocarpaceae, Taxaceae, Cephalotaxaceae, Taxodiaceae, and Araucariaceae [4]. The latter is categorized under the order Pinales (Coniferales). Taxonomically, the family is classified into four genera, *Araucaria* Juss., *Agathis* Salisb., *Columbea* Salisb., and *Wollemia* W.G. Jones, K.D. Hill, and J.M. Allen [5].

*Agathis* is an interesting genus comprising about 18 species, widely distributed in the Philippines, New Zealand, Australia, New Guinea, Melanesia, and Malaysia. Various phytochemical classes have been reported in *Agathis* species, including essential oils, fatty acids, flavonoids (in particular, the bioflavonoids), tannins, phenolics, and diterpenes. Moreover, the extracts and essential oils of *Agathis* species revealed several bioactivities, such as antimicrobial, anti-inflammatory, and cytotoxic properties [5]. One of the most ancient *Agathis* trees, *Agathis robusta* (C. Moore ex F. Muell.) F. M. Bailey, is primarily famed for the common names of Kauri pine, smooth-barked Kauri or Queensland Kauri, and it is native to New Guinea, the Bismarck Archipelago, and Queensland. It is a long-lived monoecious species characterized by a straight trunk with smooth brownish bark, which grows up to 50 m in height and 3 m in diameter [6,7].

Few phytochemical studies have been conducted on *A. robusta*. These studies have mainly focused on the investigation of the essential oil composition and diterpenoid content [7,8,9,10,11,12]. Besides, only one phytochemical analysis was conducted for the ethanolic leaf extract of *A. robusta*, resulting in the isolation and identification of six compounds; agathisflavone, 7″-*O*-methyl-agathisflavone, cupressuflavone, rutin, shikimic acid, and (2S)-1,2-Di-*O*-[(9Z,12Z,15Z)-octadeca-9,12,15-trienoyl]-3-*O*-*β*-d-galactopyranosylglycerol [13]. Moreover, few studies explored the biological activities of this species, revealing that the leaf ethanolic extract possesses a considerable anti-inflammatory activity, while the essential oil has an antibacterial effect [7,14]. Recently, the bark essential oil of *A. robusta* has been shown to have antiviral activity against SARS-CoV2 [12]. However, no previous works about the total phytochemical composition of *A. robusta* bark extract nor its possible biological activities have been performed. Therefore, the aim of the present work was to profile the chemical composition of *A. robusta* ethanolic bark extract (ARBE) and to examine its potential protective effect in RIRI using in silico and in vivo validation studies.

## 2. Results

**Figure 1:**
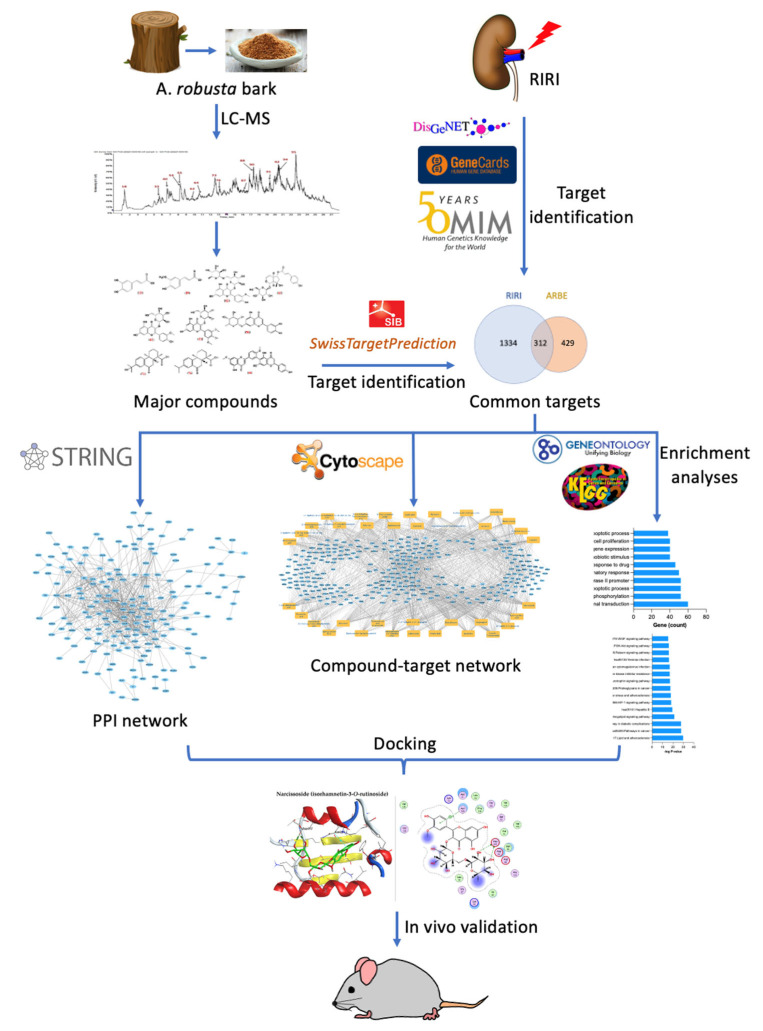
Flowchart of the workflow showing the experimental design of this study, including phytochemical, network pharmacological, and experimental studies for the investigation of the effect of ARBE in RIRI.

This study included a rational experimental design (Figure 1), involving the identification of the chemical composition profile of ARBE, using Liquid Chromatography combined with Electrospray Ionization Tandem Mass Spectrometry (LC-ESI-MS/MS). The identified compounds were subjected to in silico strategies, including network pharmacology as a modern method to investigate the interactions of active components with a disease’s molecular targets, followed by molecular docking analysis. The in vivo validation comprised a rat model of RIRI followed by functional and immunohistochemical analyses.

### 2.1. LC-ESI-MS/MS Profile

**Figure 2:**
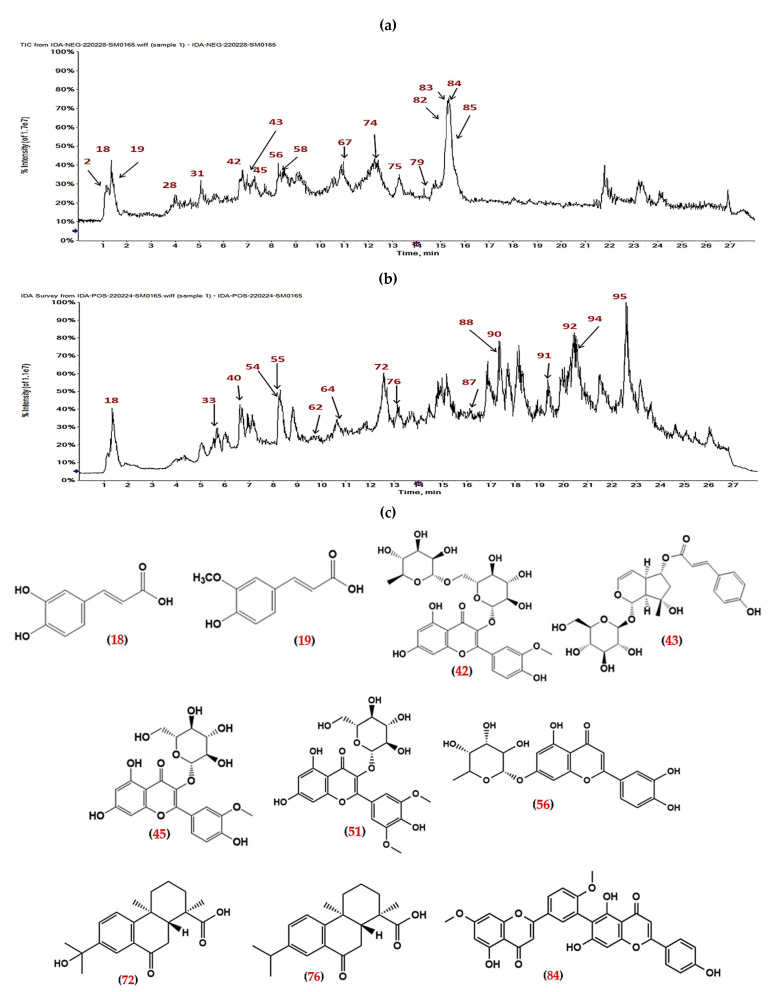
LC-MS total ion chromatograms of ARBE. (**a**) The TIC in negative ion mode. (**b**) The TIC in positive ion mode. (**c**) The structure of some LC-MS identified major components of ARBE. Numbers in red (in (**a**–**c**)) are related to Table 1.

In the present work, the phytochemical profiling of *A. robusta* bark by LC-ESI-MS/MS (negative and positive mode ESI) revealed the presence of 95 secondary metabolites, including mainly diterpenoid acids, biflavonoids, procyanidins, phenolic acids, and their derivatives, in addition to other classes, such as carboxylic acids, nucleobases, amino acids, stilbene and their derivatives, coumarins, flavonoid aglycones, flavonoid glycosides, iridoids, and norlignan. The major compounds (37 compounds) were determined on the basis of their peak area values (Table S1). Table 1 represents the data of LC-ESI-MS/MS analysis of *A. robusta* bark metabolites and Figure 2 displays the total ion chromatogram (TIC) of the extract in positive and negative mode ESI. The mass spectra of the identified compounds are depicted in Figure S1.

### 2.2. The Network Pahrmacology Analysis

#### 2.2.1. Pharmacokinetics of ARBE Major Compounds

To identify potential bioactive components, the 37 major compounds of ARBE were screened based on their pharmacokinetics and drug-likeness properties (Table S2). Indeed, most of these compounds showed higher bioavailability scores (OB > 0.55) and complied with Lipinski’s rule of five, a rule of thumb to evaluate drug likeness. However, a few compounds did not meet the screening criteria perfectly, including the procyanidins, which are known to have multiple biological activities [63]. Therefore, we included all the major ARBE compounds in our study for a comprehensive analysis.

#### 2.2.2. Molecular Targets of ARBE Major Compounds

To identify the molecular targets associated with the major constituents of ARBE, the Swiss Target Prediction database was utilized. This analysis resulted in 741 targets after the removal of duplicates (Table S3).

#### 2.2.3. Molecular Targets of ARBE Associated with RIRI

**Figure 3:**
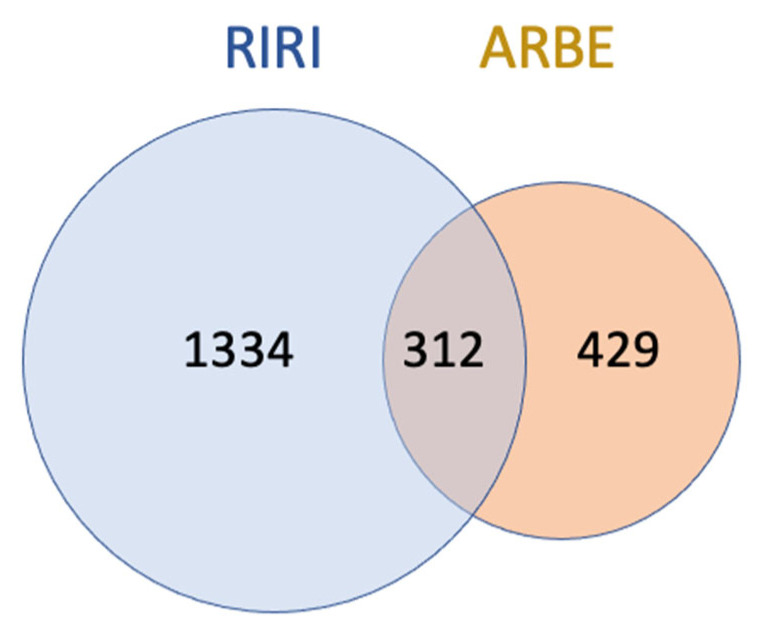
Overlapping molecular targets between RIRI and major compounds of ARBE. ARBE, Agathis Robusta bark extract; RIRI, renal ischemia reperfusion injury.

To retrieve the molecular targets associated with RIRI, three disease-related databases, i.e., DisGeNeT, GeneCards, and OMIM, were accessed. The resulting targets were 1745 and were cut down to 1646 after removal of duplicates (Table S4). Among these targets, 312 (Table S5) overlapped with the 741 targets associated with ARBE major compounds (Figure 3).

#### 2.2.4. Protein–Protein Interaction (PPI) Network of the 312 Disease-Compounds Targets

**Figure 4:**
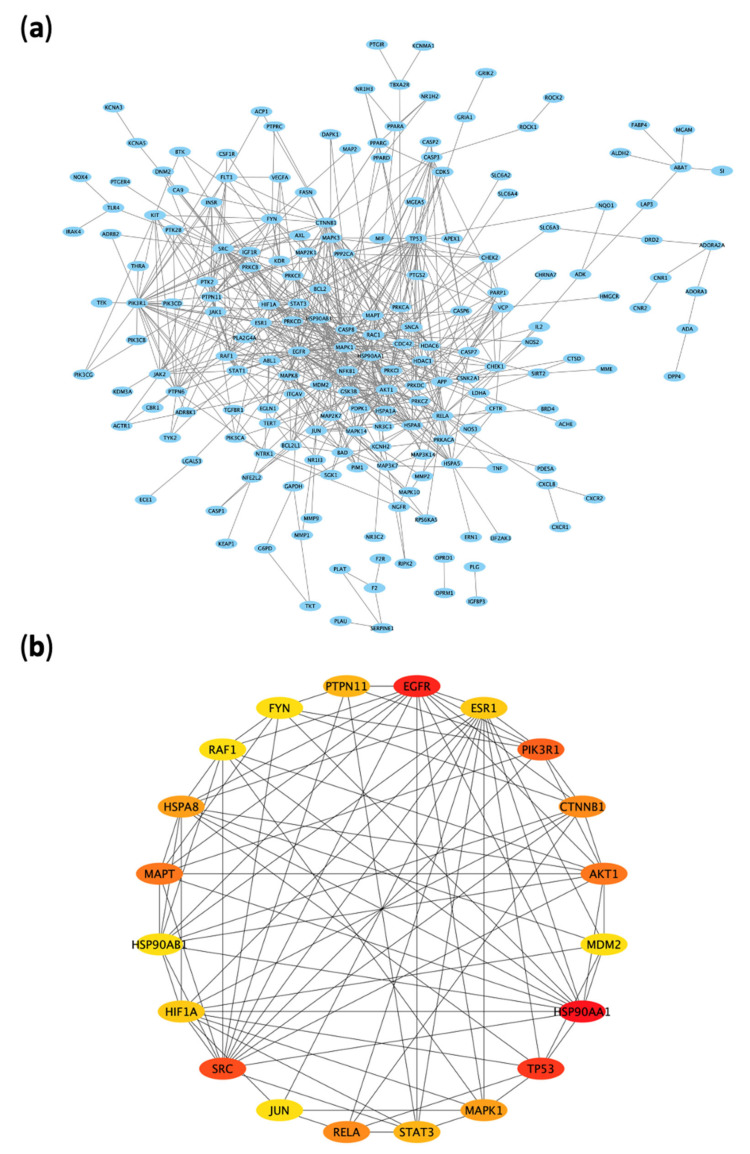
Protein–protein interaction (PPI) network of ARBE molecular targets associated with RIRI. (**a**) PPI network. (**b**) Top 20 targets in the PPI network based on degree value.

To reveal the potential molecular mechanisms of ARBE to protect from RIRI, a network of the experimentally validated PPIs of the 312 disease-compounds common targets was constructed in STRING database (Figure 4a, PPI). This network contained 312 nodes connected by 598 edges. Then, we ranked the key essential targets in the network based on their degree value, i.e., the number of connecting edges. Indeed, a higher degree value could pinpoint a more relevant target in the network. The top three targets included HSP90AA1 (degree = 42), EGFR (degree = 39), and TP53 (degree = 33). The top 20 targets with their degree value are shortlisted in Table S6 and shown in Figure 4b.

#### 2.2.5. Top ARBE Compounds Associated with RIRI Targets

**Figure 5:**
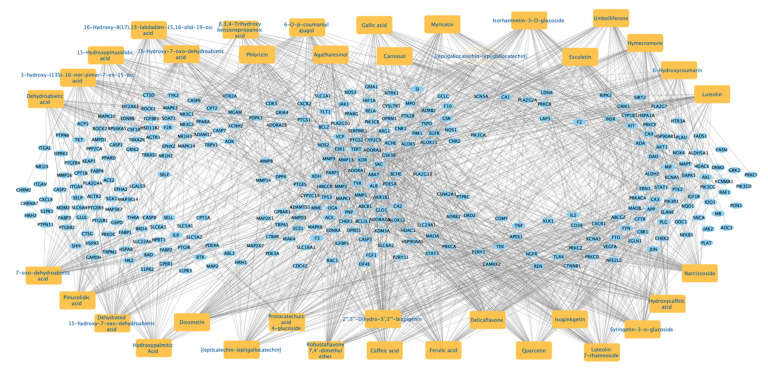
*Agathis Robusta* bark extract (ARBE) compounds—RIRI targets network.

To determine the most significant compounds of ARBE associated with the 312 RIRI targets, we constructed a compound-target network in Cytoscape (Figure 5). Then, we ranked the compounds based on degree value (Table 2).

#### 2.2.6. Gene-Ontology (GO) and KEGG Pathway Enrichment Analysis of 312 Common Targets

**Figure 6:**
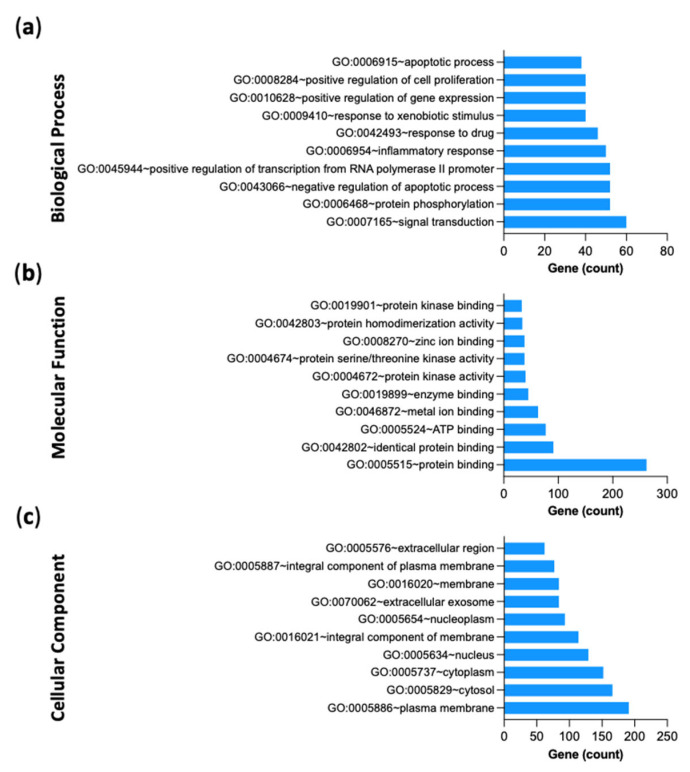
Gene Ontology (GO) analyses. (**a**) Biological process. (**b**) Molecular function. (**c**) Cellular component.

To verify the relevant biological and functional characteristics of the 312 disease-compound common targets, GO enrichment analysis was performed in biological processes (BP), molecular functions (MF), and cellular components (CC) based the number of targets that were enriched in those categories (Figure 6a–c). Top BP included signal transduction, inflammatory response and apoptotic process; Top MF included protein binding, enzyme binding, and protein kinase activity; Top CC included plasma membrane, cytoplasm, and nucleus. Detailed information of GO analyses is shown in Supplementary Tables S7–S9.

**Figure 7:**
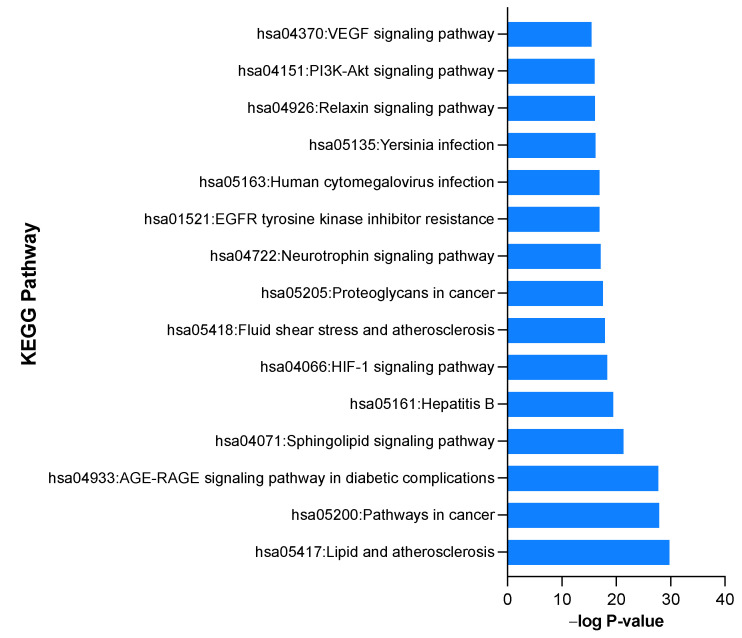
Kyoto Encyclopedia of Genes and Genomes (KEGG) pathway enrichment analyses.

To recognize the possible pathways involved in the protective effects of ARBE in RIRI, KEGG pathway enrichment analysis of the 312 disease-compounds common targets was performed (*p* < 0.05). Top enriched pathways included lipid and atherosclerosis, HIF-1 signaling pathway, and PI3K-Akt signaling pathway (Figure 7). KEGG pathway results are presented in detail in Table S10.

### 2.3. Molecular Docking Study

To examine the interaction of ARBE compounds with key RIRI molecular targets, we performed a molecular docking analysis. Ten compounds (Table 3), selected according to their peak area values and score based on degree value (Table 2), and the three top targets of RIRI (HSP90, EGFR, and p53) were included in the analysis.

Heat shock protein HSP 90-Alpha in complex with T5M (PDB code: 2XHX) [64], epidermal growth factor receptor (EGFR) tyrosine kinase in complex with erlotinib (PDB code: 1M17) [65], and cellular tumor antigen P53 (PDB code: 3Q01) [66] were implemented to provide insight on binding affinity of ARBE with the active pockets of the targeted proteins.

#### 2.3.1. Docking with Heat shock Protein HSP 90-Alpha

**Figure 8:**
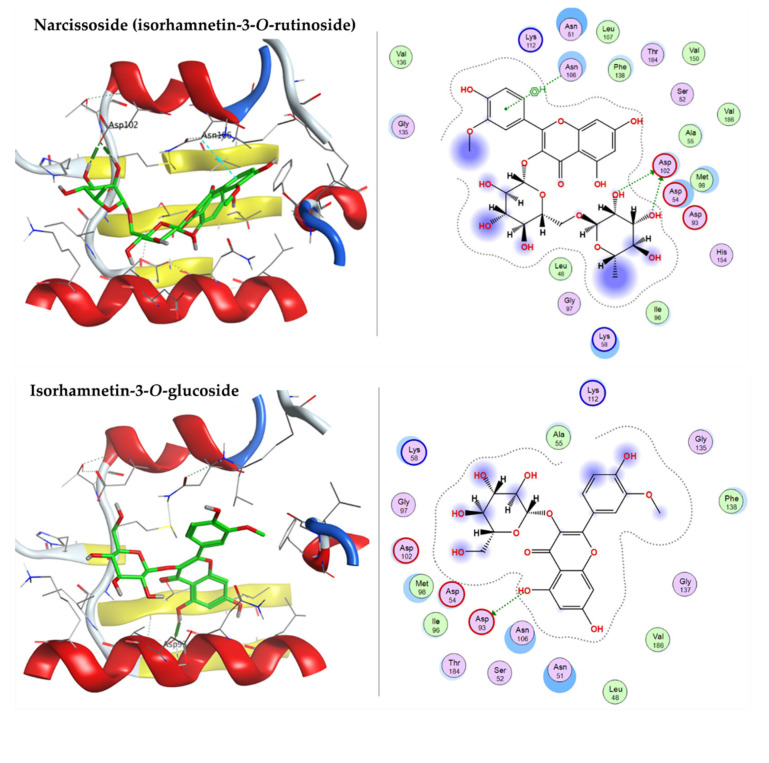

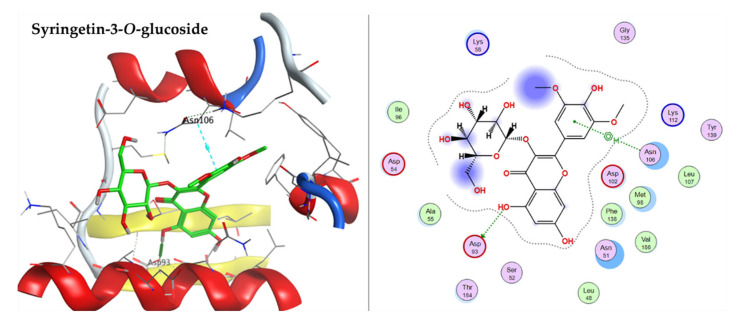
3D and 2D docking plots of narcissoside (isorhamnetin-3-*O*-rutinoside), isorhamnetin-3-*O*-glucoside, and syringetin-3-*O*-glucoside, respectively, on HSP90A (PDB code: 2XHX).

The adenine-binding site of HSP90-A (PDB code: 2XHX) is a hydrophobic pocket. Re-docking the co-crystallized ligand, 2-tert-butyl-4-(1,3-dihydro-2H-isoindol-2-ylcarbonyl)phenol (T5M) revealed the reliable active site as the RMSD is 1.2702 Å and the energy score (S) is −6.4559 kcal/mol. Docking simulations of the selected components of ARBE inside the HSP90-A active pocket exhibited that 7-oxo-dehydroabietic acid, caffeic acid, narcissoside (isorhamnetin-3-*O*-rutinoside), isorhamnetin-3-*O*-glucoside, syringetin-3-*O*-glucoside, 15-hydroxy-7-oxo-dehydroabietic acid, 6-*O*-*p*-coumaroyl ajugol, luteolin 7-rhamnoside, robustaflavone 7,4’-dimethyl ether, and ferulic acid oriented within the active pocket with reasonable docking energy score, ranging from −8.4051 to −5.0937 kcal/mol (Table 3). The docked components showed not only H-bond interactions but also arene-H bond interactions with the crucial amino acid residues described in detail in Table 3. Narcissoside (isorhamnetin-3-*O*-rutinoside), isorhamnetin-3-*O*-glucoside, and syringetin-3-*O*-glucoside have lower S scores than the co-crystallized ligand (T5M), indicating better binding affinity within the pocket (Figure 8).

#### 2.3.2. Docking with Epidermal Growth Factor Receptor (EGFR)

**Figure 9:**
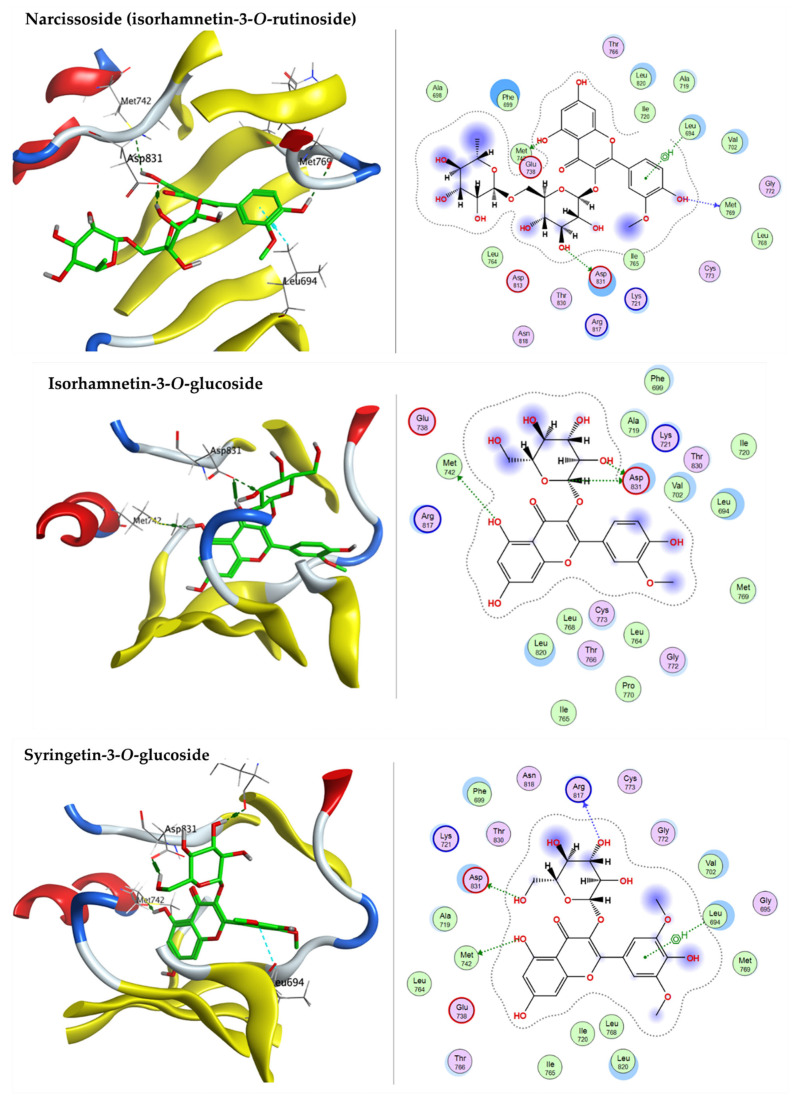
3D and 2D docking plots of narcissoside (isorhamnetin-3-*O*-rutinoside), isorhamnetin-3-*O*-glucoside, and syringetin-3-*O*-glucoside, respectively, on EGFR (PDB code: 1M17).

Docking studies reveal that 7-oxo-dehydroabietic acid, caffeic acid, narcissoside (Isorhamnetin-3-*O*-rutinoside), isorhamnetin-3-*O*-glucoside, syringetin-3-*O*-glucoside, 15-hydroxy-7-oxo-dehydroabietic acid, 6-*O*-*p*-coumaroyl ajugol, luteolin 7-rhamnoside, robustaflavone 7,4’-dimethyl ether, and ferulic acid on epidermal growth factor receptor (EGFR) tyrosine kinase (PDB code: 1M17) reached the binding site of the enzyme. In comparison to the co-crystallized ligand (AQ4: erlotinib), the docked components showed a good binding affinity, where the docking energy score ranged from −9.0112 to −5.1249 kcal/mol. The components confirmation within the active pocket stabilized by the H-bond interaction with the crucial amino acid residues. Table 4 shows the detailed amino acid residues involved in the interactions with the docked components. One of hydroxyl group of narcissoside (isorhamnetin-3-*O*-rutinoside), isorhamnetin-3-*O*-glucoside, and syringetin-3-*O*-glucoside acts as an anchor, forming an H-bond interaction with acidic ASP831. This is allowed the rest of the component structure to fill the active site properly, forming more H-bond interactions with MET742, GLN767, and MET769 in addition to a *pi*-H bind interaction with LEU694 (Figure 9).

#### 2.3.3. Docking with Cellular Tumor Antigen P53

**Figure 10:**
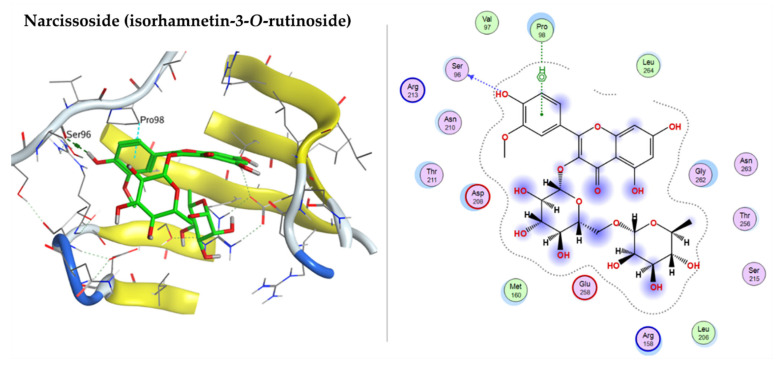

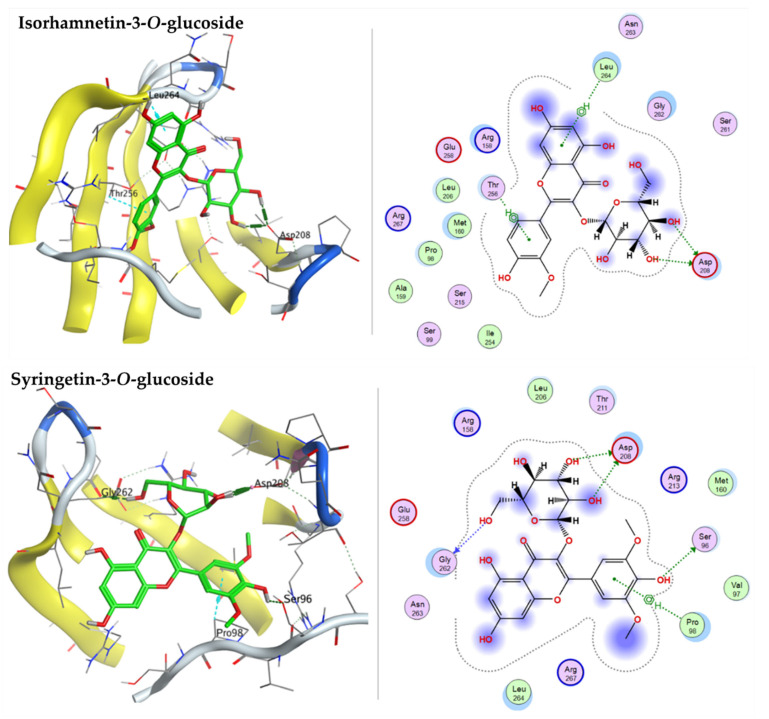
3D and 2D docking plots of narcissoside (Isorhamnetin-3-*O*-rutinoside), isorhamnetin-3-*O*-glucoside, and syringetin-3-*O*-glucoside, respectively, on P53 (PDB code: 3Q01).

The P53 active pocket is lined with the following amino acid residues: PRO98, SER99, ARG158, ALA159, MET160, LEU206, ASP208, ARG213, SER215, ILE254, ILE255, THR256, GLU258, GLY262, LEU264, and ARG267. 7-Oxo-dehydroabietic acid, caffeic acid, narcissoside (isorhamnetin-3-*O*-rutinoside), isorhamnetin-3-*O*-glucoside, syringetin-3-*O*-glucoside, 15-hydroxy-7-oxo-dehydroabietic acid, 6-*O*-p-coumaroyl ajugol, luteolin 7-rhamnoside, robustaflavone 7,4′-dimethyl ether, and ferulic acid were docked into P53 (PDB code: 3Q01) with a good binding affinity to the active site, with the docking energy score ranging from −7.1544 to −4.5554 kcal/mol. The components confirmation within the active pocket stabilized by H-bond interaction with the amino acid residues. Table 5 shows the detailed amino acid residues involved in the H-bond and *pi*-H bond interactions with the docked components. Narcissoside (isorhamnetin-3-*O*-rutinoside), isorhamnetin-3-*O*-glucoside, and syringetin-3-*O*-glucoside have good binding interactions with the amino acid residues of the active pocket, represented on Figure 10.

### 2.4. In Vivo Validation

**Figure 11:**
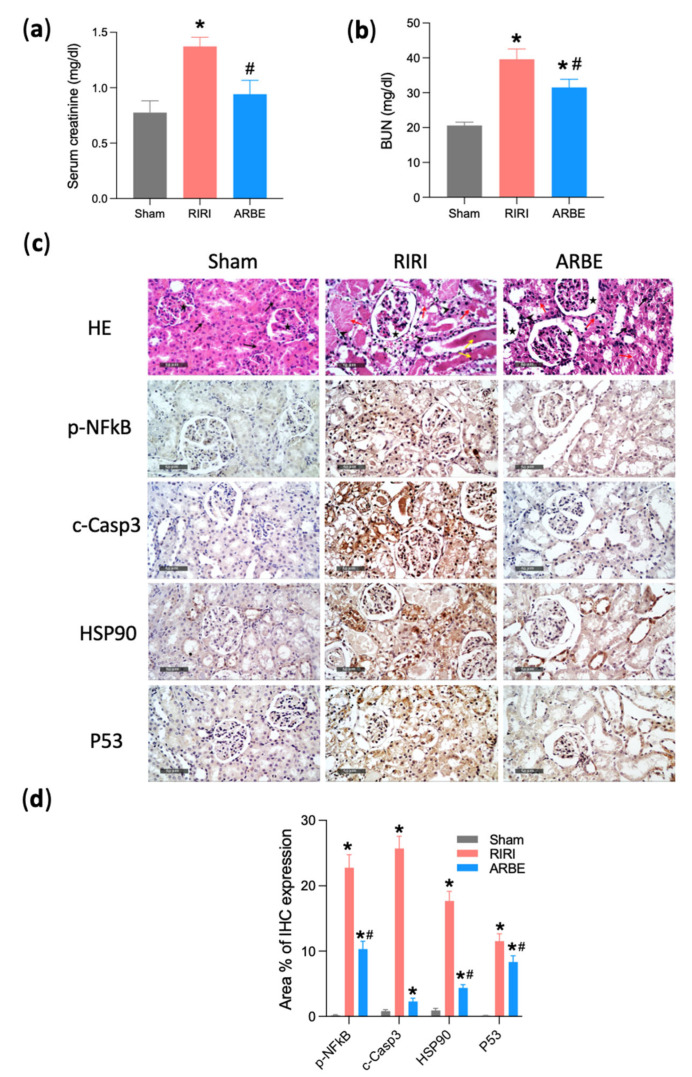
In vivo validation of the protective effect of ARBE against RIRI. (**a**) Serum creatinine. (**b**) Blood urea nitrogen (BUN). (**c**) Microscopic examination and immunohistochemical studies (Scale bar, 50 μm). (**d**) Quantification of immunohistochemical (IHC) expression. * *p* < 0.05 vs. sham, # *p* < 0.05 vs. RIRI.

To validate our in silico findings, we next tested whether ARBE is nephroprotective in an RIRI model in rats. RIRI in rats resulted in a decline in kidney function as indicated by increased serum creatinine and blood urea nitrogen, while pretreatment with ARBE improved kidney function (Figure 11a*,*b). Histopathological studies (Figure 11c) of the kidneys showed structural and pathological changes after RIRI, which were mitigated in the ARBE-pretreated animals. In Figure 11c, HE staining, sham kidneys demonstrated normal organized histological features of renal parenchyma with abundant records of apparent intact renal corpuscles (star), renal tubular segments with almost intact tubular epithelium (arrow), as well as intact vasculatures without abnormal morphological changes records; RIRI kidneys showed sever diffuse tubular epithelial loss and necrotic changes of different nephron segments (red arrow) alternated with abundant figures of degenerated pyknotic tubular epithelium with marked tubular dilatation. Significant congested glomerular tuft capillaries with significant dilatation of Bowman’s spaces (star) with focal interstitial extravasation of blood. Marked records of intraluminal eosinophilic casts (yellow arrow) with moderate interstitial mononuclear inflammatory cells infiltrates (arrow head); kidneys of ARBE-pretreated rats showed significant protective efficacy on renal tubular epithelium with persistent moderate records of tubular epithelial degenerative changes (red arrow), alternated with relative higher records of apparent intact tubular segments (black arrow). Mild persistent records of tubular dilatations were shown as well as persistent dilatation of bowman*’*s spaces (star). Moreover, there were minimal records of interstitial inflammatory cells infiltrate (arrow head) and intraluminal casts with intact vasculatures. In the immunohistochemical analysis, in comparison to the vehicle, ARBE pre-treatment in rats with RIRI significantly dampened renal inflammation and apoptosis as indicated by reducing p-NfKB and cleaved caspase-3 expression, respectively (Figure 11c,d).

Next, we examined whether these nephroprotective activities of ARBE in RIRI are due to modulating the top RIRI molecular targets which were identified by network analysis and confirmed by docking. Therefore, we assessed the renal expression of two of these targets, i.e., HSP90 and P53, which have been shown to contribute to kidney dysfunction, inflammation, and cell death in previous studies [67,68]. Indeed, RIRI led to increased expression of both proteins in rat kidney, yet, pretreatment with ARBE attenuated this effect (Figure 11c*,*d).

## 3. Discussion

Based on phytochemical characterization followed by network pharmacology, docking, and preclinical validation, we here report a potential protective effect of ARBE against RIRI. This study revealed the most biologically significant components of ARBE and their possible molecular targets and mechanisms of actions in attenuation of RIRI-induced AKI.

In the phytochemical analysis, LC-ESI-MS/MS chromatographic technique was employed to characterize the phytometabolites of ARBE. In total, 95 compounds were tentatively identified according to their MS and MS^2^ fragmentation data and retention times. The major identified chemical classes include diterpenoid acids, such as 7-oxo-dehydroabietic acid, abietic acid, and pinusolidic acid; biflavonoids, such as robustaflavone 7,4′-dimethyl ether and delicaflavone; procyanidins, such as as [(epi)gallocatechin-(epi)gallocatechin] and [(epi)catechin-(epi)gallocatechin]; and phenolic acids, such as caffeic and gallic acids.

The presence of such diverse chemical components in herbs makes it complex to assess the potential therapeutic action by the single component-single target paradigm [69]. Therefore, network pharmacology-based strategy could be a potential successful tool in this context. This approach utilizes a computational action plan to uncover the possible component-target-disease associations, and has been recognized as an efficient method in multiple previous studies [69,70,71,72]. Applying network pharmacology in this study led to the identification of 312 RIRI-related molecular targets that can be potentially aimed at by major components of ARBE. Of those targets, the most relevant hubs, based on experimentally validated PPIs, included molecular chaperones, i.e., HSPs, which regulate protein folding, intracellular transport, and repair or degradation [73]. Proteins involved in signaling pathways, cell survival, and cell division, e.g., EGFR and P53, were also included as significant targets [74,75].

Docking is a useful tool to predict the chemical reactivity of compounds towards molecular targets [76]. In this study, ten ARBE compounds with high peak area and degree value scores showed substantial binding with the top three RIRI targets in the experimentally validated PPI network, i.e., HSP90A, EGRF, and P53.

The molecular chaperone HSP90A is a homodimeric protein [77]. The HSP90A structure consists of three domains: N-terminal domain (N-domain), middle domain (M-domain), and C-terminal domain (C-domain). The N-domain, the catalytic domain, binds with ATP. M-domain associates both N- and C-domains, while the N-domain connects with its partner domain in the other subunit to form the dimer. The HSP90 chaperone cycle includes the turnover of ATP to ADP through the ATPase action in the N-terminal domain. The ATP binding site has been described by NMR and/or X-ray diffraction by the binding modes of a number of reported HSP90A inhibitors [78]. It was reported that resorcinol-bearing compounds are considered as lead compounds for discovering inhibitors or modulators of HSP90 [64,79]. The docked components have hydroxyl/phenolic groups exhibited interactions with the amino acids present in the adenine-binding site of HSP90A, proposing that ARBE may be effective in prevention or treatment of a diseases mediated by HSP90A.

The EGFR kinase domain (EGFR) has a characteristic bilobate-fold. The *N*-terminal domain is formed from mostly *β*-strands and one α-helix, whereas the larger *C*-terminal domain is formed from mostly α -helices. The two domains are separated by a cleft similar to those in which ATP, ATP analogues, and ATP inhibitors have been found to bind [65]. Docked components ARBE showed promising interactions through fitting into EGFR adenine pocket.

Cellular tumor antigen P53 (PDB code: 3Q01) was crystallized as a homodimer [66]. The resolved P53 protein structure has no co-crystallized ligand, so both Computed Atlas for Surface Topography of Proteins (CASTp) [80] and Site Finder module in MOE 2019.0102 were used to find potential 3D pockets for P53 protein. Molecular docking of the components within P53 active pocket revealed that they exhibited promising binding energies with potential activity on P53.

Computational analysis without experimental validation could be non-sufficient and meaningless. Thus, a preclinical model of RIRI was used in this study to examine the potential activity and mechanism of ARBE. This experiment revealed a nephroprotective effect of ARBE in RIRI by diminishing renal inflammation and apoptosis. These effects were due to downregulation of both HSP90 and P53 by ARBE. Indeed, previous studies showed that both HSP90 and P53 are upregulated after RIRI resulting in inflammation, oxidative stress, cell death, and structural changes. Both genetic and pharmacological inhibition of these proteins in RIRI are associated with better outcomes [67,68,81,82]. For instance, AT13387, an HSP90 inhibitor, ameliorated RIRI by abolishing Toll-like receptor 4 (TLR4)-mediated NF-κB activation [67]. Furthermore, intravenous injection of synthetic siRNA to p53 after ischemic injury protected both proximal tubule cells and kidney function in rats [83]. In a clinical study (ClinicalTrials.gov identifier: NCT00802347), a single systemic administration of QPI-1002, a siRNA-based p53 inhibitor, reduced the incidence of delayed graft function in deceased donor allograft recipients by downregulating p53 following reperfusion [84].

## 4. Materials and Methods

### 4.1. Plant Material and Extraction

*A. robusta* fresh bark (50 years old cultivated tree) was collected in July 2021 from El-Orman Botanical Garden, Giza, Egypt. The plant identification was confirmed by Dr. Therese Labib Youssef, ExManager and Taxonomist at Orman Botanical Garden, Giza, Egypt. A voucher sample [Reg. No. (ZU-Ph-Cog-0200)] was deposited within the Herbarium of the Pharmacognosy Department, Faculty of Pharmacy, Zagazig University.

For extraction, the dried powdered bark (280 g) was macerated with 70% ethanol (3 × 1 L). The extract was evaporated at reduced pressure to afford 80 g of viscous residue.

### 4.2. LC-ESI-MS/MS Analysis

The bark extract was reconditioned in (Water: Methanol: Acetonitrile, 50:25:25 *v/v*), and analyzed by LC-ESI-MS/MS using ExionLC (High flow LC, Sciex^®,^ Framingham, MA, USA), coupled with TripleTOF 5600+Time-of-Flight (IDA Acquisition, Sciex^®^) and Analyst TF 1.7.1 (LC-Triple TOF control, Sciex^®^). The injection concentration and volume were 2.5 µg/µL and 10 µL, respectively. The pre-column used consisted of In-Line filter disks (0.5 µm × 3.0 mm, Phenomenex^®^_,_ Torrance, CA, USA), while the used column was X select HSS T3 (2.5 µm, 2.1 × 150 mm, Waters^®^_,_ Milford, MA, USA) and the column temperature was set at 40 °C. The flow rate was 0.3 mL/min, and the elution was carried out using a buffer system of 1% methanol in 5 mM ammonium formate at pH 3 as solvent A for positive mode, at pH 8 as solvent B for negative mode and 100% of acetonitrile as solvent C. Gradient elution was carried out as follows: 90% solvent A or B and 10% of solvent C were injected for 20 min, then turned to 10% of solvent A or B to 90% of solvent C for the next 5 min, and finally, by the starting elution mixture was applied for the last 3 min. PeakView was employed for peaks extraction from the total ion chromatogram (TIC), on the basis of that the peaks should possess a signal-to-noise ratio greater than 5 (non-targeted analysis); in addition, the peak intensities of the sample-to-blank should be greater than 3. The interpretation of data was achieved using a ReifycsAbf (Analysis Base File) Converter for Wiff file conversion (Reifycs^®^, Tokyo, Japan) and MS-DIAL 4.6 (RIKEN^®^ Tokyo, Japan). The compounds were tentatively identified according to their retention time, MS, and MS^2^ fragmentation using PeakView^TM^ software version 2.1, and the peak area values were calculated using the XIC Manager in this software. For each identified compound, extracted ion chromatograms (XICs) were automatically produced and compared to a user-defined threshold [85].

### 4.3. Network Pharmacology

#### 4.3.1. Acquisition of Pharmacokinetics and Associated Targets of ARBE Major Compounds

SMILES of the major compounds of ARBE were obtained from PubChem (https://pubchem.ncbi.nlm.nih.gov/, accessed on 25 June 2022) database or using ChemDraw v20.0.0.41 (PerkinElmer Informatics, Inc., UK). The pharmacokinetics of these compounds were acquired by using the SwissADME web tool (http://www.swissadme.ch/, accessed on 25 June 2022) [86].

The molecular targets linked to the identified major compounds of AR were predicted using the SwissTargetPrediction (http://www.swisstargetprediction.ch/, accessed on 1 July 2022) database [87].

#### 4.3.2. Identification of RIRI-Associated Targets

The molecular targets associated with RIRI were obtained from DisGeNeT (https://www.disgenet.org/search, accessed on 24 June 2022) [88], GeneCards (https://www.genecards.org/, accessed on 24 June 2022) [89,90], and Online Mendelian Inheritance in Man (OMIM, https://www.omim.org/, accessed on 24 June 2022) databases [91].

#### 4.3.3. Construction of PPI and Compound-Target Networks

The common overlapping targets between ARBE major compounds and RIRI targets were identified in Microsoft Excel and represented as a Venn diagram.

Protein–protein interactions (PPI) network of the intersected targets between the AR compounds and RIRI-related targets was constructed using the STRING database Version 11.5 (https://string-db.org/, accessed on 2 July 2022) [92]. A confidence level of >0.4 in protein interactions was applied, and only experimentally validated PPI were included. A compound-target network was also constructed between the major compounds of AR and the intersected targets.

The Cytoscape 3.9.1 software program (NIGMS, USA) [93] was utilized to visualize the networks. The key essential targets and top compounds were ranked based on degree by applying the CytoHubba plugin contained in Cytoscape [94].

#### 4.3.4. The GO Analysis and KEGG Pathway Enrichment

A database for Annotation, Visualization and Integrated Discovery (David database, https://david.ncifcrf.gov/tools.jsp, accessed on 3 July 2022) [95] was employed to perform GO analysis and KEGG pathway enrichment. A *p*-value < 0.05 was used as a cutoff and was corrected using a false discovery rate (FDR) error control technique.

### 4.4. Molecular Modelling

#### 4.4.1. Molecular Docking Study

Molecular docking studies of the following components: 7-oxo-dehydroabietic acid, caffeic acid, narcissoside (Isorhamnetin-3-*O*-rutinoside), isorhamnetin-3-*O*-glucoside, Syringetin-3-*O*-glucoside, 15-hydroxy-7-oxo-dehydroabietic acid, 6-*O*-*p*-coumaroyl ajugol, luteolin 7-rhamnoside, robustaflavone 7,4′-dimethyl ether, and ferulic acid were performed to evaluate their binding affinity with the targeted active sites of HSP90A, EGFR, and P53 proteins. Molecular Operating Environment MOE version 2019.0102 software (Chemical Computing Group, Montreal, CA) [96] was used for the docking studies. The exploited docking placement methodology is triangle matcher. Each ligand was allowed to be flexible, while the protein structure was kept rigid. The scores of the docking energy for the best-fitted poses of the components with the protein active pocket were recorded.

#### 4.4.2. Protein Preparation

The crystal structures of HSP90A (PDB code: 2XHX/2.80 Å), EGFR (PDB code: 1M17/2.60 Å), and P53 (PDB code: 3Q01/2.10 Å) were obtained from the Protein Data Bank (http://www.rcsb.org) [97] (Table S11). The crystallized water molecules and repeated chains were deleted from the protein complexes. The structures of HSP90A, EGFR, and P53 were prepared individually, using the MOE Quick Preparation protocol using the Amber10: EHT forcefield.

#### 4.4.3. Ligand Preparation

7-Oxo-dehydroabietic acid, caffeic acid, narcissoside (isorhamnetin-3-*O*-rutinoside), isorhamnetin-3-*O*-glucoside, syringetin-3-*O*-glucoside, 15-hydroxy-7-oxo-dehydroabietic acid, 6-*O*-*p*-coumaroyl ajugol, luteolin 7-rhamnoside, robustaflavone 7,4′-dimethyl ether, and ferulic acid were drawn through the Chemdraw^®^ (PerkinElmer Informatics, Inc., UK), then transferred to the MOE using smiles strings. The energy of the components was minimized with root mean square (RMS) gradient 0.1 kcal/mol/Â^2^ and finally preparing a database file. The co-crystallized ligands for both HSP90A, EGFR were re-docked for the validation process of the active site. P53 has no crystallized ligand; therefore, the Computed Atlas for Surface Topography of Proteins (CASTp; http://sts.bioe.uic.edu/castp/index.html, accessed on 18 July 2022 ) server was used for the active pocket prediction [80].

The score of docking energy (S; kcal/mol) and visual inspection of both two-dimensional and three-dimensional planes of the component-targeted protein interactions were exploited for the results analysis.

### 4.5. In Vivo Experiments

#### 4.5.1. Animals, Ethical Statement, and Experimental Design

All experiments were approved by the Institutional Animal Care and Use Committee of Zagazig University (ZU-IACUC/3/F/111/2022). The procedures were carried out according to the National Institute of Health guidelines. A total of 24 Wistar rats (weighing 150–180 g) were used in this study. Prior to the experiment, all rats were allowed free access to standard diet and water and were subjected to a circadian rhythm with a 12 h day and 12 h night at an ambient temperature of 24~26 °C with 50~60% humidity for 1 week. The rats were randomly divided into three groups including a sham group (*n* = 8), a RIRI group (*n* = 8), and a RIRI group treated with ARBE (*n* = 8).

#### 4.5.2. Induction of Renal Ischemia-Reperfusion (RIRI)

Renal ischemia-reperfusion injury (RIRI) was performed as previously described [98]. Briefly, ARBE (10 mL/kg, p.o., corresponding to 400 mg/kg) or vehicle was administered twice: 180 min and 15 min prior to ischemia. Dose was chosen based on a pilot study. Rats were anesthetized with thiopental sodium (Sigma Tec, Giza, Egypt) (25 mg/kg) administered i.p. before surgery. The left kidney was accessed through abdominal incision, and a non-traumatic vascular clamp was applied to the left renal artery for 60 min. Then, the clamp was removed to induce renal reperfusion. Finally, the abdominal cavity was closed. After the 24 h reperfusion period, the animals were decapitated for sample collection. The animals in the sham group underwent abdominal incision without clamping of the left renal arteries. Blood samples were collected via venipuncture and then were centrifuged, and the serum was stored at −80 °C for further analysis. Parts of the kidneys were fixed in 10% buffered formalin for histological and immunohistochemical studies. The rest of kidneys were snap frozen in liquid nitrogen and stored at −80 °C.

#### 4.5.3. Kidney Function Assessment

Serum samples were assayed for blood urea nitrogen (BUN) and serum creatinine by using a Urea Nitrogen Kit (BioDiagnostic, Giza, Egypt) and the Creatinine Kit (BioDiagnostic, Giza, Egypt), respectively, according to the manufacturer’s protocol.

#### 4.5.4. Histopathology

Kidney tissue samples were fixed in 10% neutral buffered formalin for 72 h. Samples were processed in serial grades of ethanol, cleared in Xylene, then infiltrated and embedded into Paraplast tissue embedding media. Next, 5 μm thick serial sections were cut by rotatory microtome for demonstration of renal parenchyma in different samples and mounted on glass slides. Tissue sections were stained by Hematoxylin and Eosin as a standard staining method for blinded light microscopic examination by an experienced histologist.

#### 4.5.5. Immunohistochemistry

Five µm thick paraffin embedded tissue sections were prepared. Deparaffinized retrieved tissue sections were treated by 0.3% H_2_O_2_ for 20 Mins. Then, they were incubated with anti-Cleaved Caspase-3 (GB11532, service bio. (Wuhan, China)—1: 300), Anti-HSP 90α Antibody (sc-515081, Santa cruz Biotechnology, Inc., CA, USA—1:100), anti p-NFκB p65 antibody (GTX54672, GeneTex Inc. (Hsinchu, Taiwan)—1:100), and anti P53 (Abcam (Cambridge, UK)-ab131442—1:100) overnight at 4 °C. Tissue sections were washed out by PBS followed by incubation with secondary antibody HRP Envision kit (DAKO) for 20 min, and then washed out and incubated with diaminobenzidine (DAB) for 15 min. Next, they were washed by PBS and counter stained with hematoxylin, dehydrated and cleared in xylene, then cover slipped for microscopic examination.

Histological analysis was performed according to a previous study [99]. At least six non-overlapping fields were randomly selected and scanned from kidney tissue sections of each sample for the determination of relative area percentage of immunohistochemical expression levels of Cleaved Caspase 3, p-NFkB, HSP90, and P53 in immunohistochemically stained sections. All light microscopic examinations and data were obtained by using a Leica Application module for histological analysis, attached to a Full HD microscopic imaging system (Leica Microsystems GmbH, Wetzlar, Germany).

### 4.6. Statistical Analysis

GraphPad Prism version 9.4.1 (CA, USA) was used for the statistical analyses, and the results are presented as the means ± standard error of the mean (SEM). Comparisons between the groups were performed using the one-way ANOVA and Tukey’s multiple comparison test; significance was accepted at *p* < 0.05.

## 5. Conclusions

Overall, our findings, using phytochemical, in silico network, and docking approaches, as well as further in vivo preclinical validation, show that ARBE could protect from RIRI by combating inflammation and apoptosis. Mechanistically, this might be due to the downregulation of HSP90 and P53. Yet, the effect of ARBE on other molecular targets cannot be excluded. Further drug discovery and preclinical and clinical studies are warranted on certain major components of ARBE, e.g., syringetin-3-*O*-glucoside and narcissoside, given their predicted interaction with multiple RIRI-related targets, in particular the hubs. These compounds may represent a base for the generation of new molecules that could be utilized as nephroprotective agents against RIRI.

## Figures and Tables

**Table 1:** Metabolites identified in ARBE using LC-ESI-MS/MS in negative and positive ionization modes.

No.	Rt.	[M+H]^+^	[M−H]^−^	MS^2^ Fragments (*m/z*)	Tentative Identification	Class	Ref.
1.	1.065		191.056	173, 85	Quinic acid	Cyclohexanecarboxylic acid	[15]
2.	1.117		169.014	125	**Trihydroxy benzoic acid (gallic acid)** *	Phenolic acid	[16]
3.	1.143		117.019	73, 99	Succinic acid	Omega-dicarboxylic acid	[17]
4.	1.144		153.019	109	Dihydroxybenzoic acid (protocatechuic acid)	Phenolic acid	[16]
5.	1.145		181.013	137, 113, 109	Dihydrocaffeic acid [3-(3,4-Dihydroxyphenyl)propionic acid]	Phenolic acid	[16]
6.	1.145		197.0106	179, 153,109	***β*,3,4-Trihydroxy benzenepropanoic acid** *	Phenolic acid	[18]
7.	1.147	104.107		60, 58	*N-*methyl alanine	Amino acid	[19]
8.	1.169		109.028	81	1,2-Benzenediol	Phenol dvs.	[20]
9.	1.173	104.105		71, 60, 58	Choline	Cholines	[21]
10.	1.182		137.025	93	Salicylic acid*	Phenolic acid	[16]
11.	1.185		261.045	97	Sorbitol 6-phosphate	Sugar	[22]
12.	1.186	110.060		93, 80, 69	4- Aminophenol	Phenol derivative	[23]
13.	1.211	136.061		119, 109, 92, 65	Adenine	Nucleobase (a purine derivative)	[24]
14.	1.237		315.071	153	**Protocatechuic acid 4-glucoside**	Phenolic acid hexoside	[16]
15.	1.246		173.081	129, 36	Shikimic acid	Cyclohexanecarboxylic acid	[25]
16.	1.349		167.035	152	Methoxy hydroxy benzoic acid (Vanillic acid)	Phenolic acid	[16]
17.	1.351	229.151		142, 114, 96, 70	*N*,*N*,*N*-trimethyl-l-alanyl-l-proline betaine	Stilbene	[26]
18.	1.380		179	161, 135	**Caffeic acid** *	Phenolic acid	[27]
19.	1.401		193.073	147	**Ferulic acid**	Phenolic acid	[16]
20	1.501		225.044	181, 151	Dihydrosinapic acid *	Phenolic acid	[28]
21.	1.658		161.047	117, 73	**6-Hydroxycoumarin**	Coumarin	[29]
22.	2.212	118.086		59, 58	Glycine- betaine	Alpha amino acid	[30]
23.	2.232		609.127	483, 441, 423, 305, 303, 177	**[(epi)gallocatechin-(epi)gallocatechin]** *	Procyanidin	[31]
24.	3.618		593.208	425, 407,289	[(epi)catechin-(epi)gallocatechin]	Procyanidin	[31]
25.	3.643		593.205	425, 407,289, 177	**[(epi)catechin-(epi)gallocatechin]**	Procyanidin	[31]
26.	3.684		593.208	425, 407	[(epi)catechin-(epi)gallocatechin]	Procyanidin	[31]
27.	3.815		609.240	483, 441, 423, 305, 303, 177	[(epi)gallocatechin-(epi)gallocatechin] *	procyanidin	[31]
28.	4.036		305.114	261, 221, 219, 179, 165	(epi)gallocatechin	Procyanidin	[32]
29.	4.495		609.131	483, 441, 423, 305, 303, 177	[(epi)gallocatechin-(epi)gallocatechin]*	Procyanidin	[31]
30.	4.515	122.079			Cysteine	Amino acid	[33]
31.	5.150		195.065	151, 150	Hydroxycaffeic acid*	Phenolic acid	[34]
32.	5.608		477.177	315, 300, 269	Isorhamnetin-*O*-glucoside	Flavone glucoside	[35]
33.	5.629	137.131		119, 110, 64	Hypoxanthine	Nucleobase (a purine derivative)	[36]
34.	5.854		405.191	243	3,4,3′,5′-Tetrahydroxystilbene 3′-glucoside	Stilbene glucoside	[37]
35.	5.903		177.019	149, 133, 109	**6,7-dihydroxycoumarin (esculetin)**	Coumarin	[38]
36.	6.424	611.166		449,465, 303	Quercetin-rhamnose-hexose	Flavonol glycoside	[39]
37.	6.625		267.088	252, 131	Formononetin	Isoflavone	[40]
38.	6.697		197.0091	179, 153, 135	Danshensu (*α*,3,4-trihydroxy benzenepropanoic acid)	Phenolic acid	[18]
39.	6.816		593.156	285, 284	Kaempferol-7-neohesperidoside	Flavonol glycoside	[41]
40.	6.847	465.235		303	Quercetin hexoside	Flavonol glycoside	[42]
41.	6.852		449.113	287	Okanin-4′-*O*-glucoside [2′,3′,4′,3,4-pentahydroxychalcone glucoside]*	Chalcone glucoside	[42]
42.	6.960		623.166	315, 314	Isorhamnetin-3-*O*-rutinoside (narcissoside)*	Flavonol glycoside	[41]
43.	7.011		493.200 [M-H], 539.267[M+HCOO]	493,331,313, 145	**6-*O*-*p*-coumaroyl ajugol**	Coumaroyl iridoid glycoside	[43]
44.	7.098		285.115	225	**Agatharesinol**	Norlignan	[44]
45.	7.293		477.106	315, 314	Isorhamnetin-3-*O*-glucoside*	Flavonol glycoside	[41]
46.	7.353		289.145	245	(epi)catechin*	Procyanidin	[32]
47.	7.412	449.108		287	Kaempferol hexoside	Flavonol glycoside	[45]
48.	7.451	301.107		153, 229, 257	**3, 5, 7-trihydroxy-4**′**-methoxyflavone (Diosmetin)**	Flavonols	[27]
49.	7.584	433.127		271, 85	Apigenin-*O*-hexoside	Flavonone -O-glycosides	[46]
50.	7.913		435.130	273, 167, 123	Phlorizin*	Dihydrochalcone glucoside	[47]
51.	7.942	509.099		347, 332	**Syringetin-3-*O*-glucoside**	Flavonol glycoside	[48]
52.	8.024	(331.191) M+		313, 287, 255	7-Hydroxy-8-methoxydedihydrorutaecarpine (7-Hydroxy-8-methoxy,7,8-Dehydrorutaecarpine)	Alkaloid	[49]
53.	8.092		317.029	179, 151, 137	Myricetin*	Flavone	[50]
54.	8.192	163.076		131, 103	**Umbelliferone**	Coumarin	[51]
55.	8.274	177.091		162, 149	**Hymecromone (4-methylumbelliferone)**	Coumarin	[52]
56.	8.296		431.099	285,284, 255	**luteolin 7-*O*-rhamnoside**	Flavone glycoside	[53]
57.	8.375	319.045		301, 273, 217	Steviol	Ent-kaurane diterpenoid	[54]
58.	8.397		347.187	303, 301, 285	**16-Hydroxy-8(17),13-labdadien-15,16-olid-19-oic acid**	Diterpenoid acid	[42]
59.	9.094	377.165		359	Angelol A	Coumarins	[51]
60.	9.668		541.239[M+HCOO], 495[M-H]	349,333, 163	6-*O*-*p*-coumaroyl dihydroajugol	Coumaroyl iridoid glycoside	[43]
61.	9.704		303.123	259, 285	Copalic acid*	Diterpenoid acid	[55]
62.	9.771	303.050		257, 201	Abietic acid	Diterpenoid acid	[56]
63.	10.399	301.216		283, 255	Retinoic acid	Retinoids	[57]
64.	10.934	301.217		255, 173, 147,133, 109	**Dehydroabietic acid**	Diterpenoid acid	[56]
65.	11.015	305.174		287, 259, 159	Taxifolin (dihydroquercetin)	Flavanonols	[58]
66.	11.106	287.055		269, 153	**Luteolin**	Flavones	[27]
67.	11.401		347.223	303, 301	**15-Hydroxypinusolidic acid**	Diterpenoid acid	[42]
68.	11.814	377.143		359	Isoangelol	Coumarins	[51]
69.	12.278		897.071	693, 289	(epi)gallocatechin-(epi)gallocatechin-(epi)catechin	Procyanidin	[31]
70.	12.419		577.117	289	(epi)catechin-(epi)catechin	Procyanidin	[32]
71.	12.57		305.212	261, 287	**3-hydroxy-(13S)-l6-nor-pimar-7-en-l5-oic acid** *	Diterpenoid acid	[44]
72.	12.658	331.189		203	**15-hydroxy-7-oxo-dehydroabietic acid**	Diterpenoid acid	[56]
73.	12.711		329.177	285,311	**Carnosol**	Phenolic diterpene	[55]
74.	12.788		301.0349	255, 179, 151	Quercetin*	Flavonol	[32]
75.	13.014		565.113	389	**Isoginkgetin**	Biflavonoids	[59]
76.	13.058	315.161		199, 187	7-oxo-dehydroabietic acid	Diterpenoid acid	[56]
77.	13.36		283.061	268	Chrysin-6-methyl-ether	Flavonone	[60]
78.	13.449		283.061	268,239	Physcion	dihydroxyanthraquinone	[61]
79.	14.413		331.229	287	**Pinusolidic acid** *	Diterpenoid acid	[42]
80.	14.567		271.228	253,227, 225	**Hydroxypalmitic Acid**	Fatty acid	[55]
81.	14.76		299.166	284, 255	4′-Hydroxywogonin	Trihydroxy-methoxyflavone	[29]
82.	15.382		539.168	539, 387	**2**″**,3**″**-Dihydro-3**′**,3**‴**-biapigenin**	Biflavonoids	[59]
83.	15.602		536.883	537, 385	2′,8″-Biapigenin	Biflavonoids	[59]
84.	15.618		565.114	445, 388, 403, 456, 471	**Robustaflavone 7,4**′**-dimethyl ether**	Biflavonoids	[59]
85.	15.911		537.305	537, 193, 192	**Delicaflavone**	Biflavonoids	[59]
86.	15.929	303.229		285, 257, 239	Abietic acid isomer	Diterpenoid acid	[56]
87.	16.014	301.216		283, 255, 133, 109	Dehydroabietic acid isomer	Diterpenoid acid	[56]
88.	17.120	313.179		211, 197, 185	**Dehydrated 15-hydroxy-7-oxo-dehydroabietic acid**	Diterpenoid acid	[56]
89.	17.17		363.212 [M+HCOO]	317, 159	Agarotetrol formate adduct	2-(2-phenylethyl) chromones	[62]
90	17.679	315.195		199, 187	**7-oxo-dehydroabietic acid isomer**	Diterpenoid acid	[56]
91.	19.553	303.199		285, 257, 109	Abietic acid isomer	Diterpenoid acid	[56]
92.	20.795	301.217		255, 173, 147,133, 109	Dehydroabietic acid isomer	Diterpenoid acid	[56]
93.	20.857	301.216		255, 173,133, 109	Dehydroabietic acid isomer	Diterpenoid acid	[56]
94.	20.883	301.217		255, 173, 147,133, 109	Dehydroabietic acid isomer	Diterpenoid acid	[56]
95.	22.529	315.196		297,199, 187	7-oxo-dehydroabietic acid isomer	Diterpenoid acid	[56]

* Identified also in positive mode. Bold compounds refer to the major ones.

**Table 2:** Major compounds of ARBE ranked by the Degree method.

Rank	Target Name	Score
1	Narcissoside	60
2	Isorhamnetin-3-*O*-glucoside	58
2	Syringetin-3-*O*-glucoside	58
4	6-*O*-p-coumaroyl ajugol	56
5	Luteolin 7-rhamnoside	55
6	Robustaflavone 7,4′-dimethyl ether	53
6	Ferulic acid	53
6	2″,3″-Dihydro-3′,3‴-biapigenin	53
9	3-hydroxy-(13S)-16-nor-pimar-7-en-15-oic acid	52
9	Caffeic acid	52
11	Agatharesinol	51
12	Delicaflavone	50
12	Dehydrated 15-hydroxy-7-oxo-dehydroabietic acid	50
12	Diosmetin	50
15	Esculetin	49
15	Hydroxycaffeic acid	49
17	Hymecromone	48
17	Myricetin	48
17	Isoginkgetin	48
17	16-Hydroxy-8(17)13-labdadien-1516-olid-19-oic acid	48
21	Quercetin	47
21	[(epi)gallocatechin-(epi)gallocatechin]	47
21	Luteolin	47
21	Umbelliferone	47
25	15-hydroxy-7-oxo-dehydroabietic acid	46
25	7-oxo-dehydroabietic acid	46
27	Phlorizin	45
27	Protocatechuic acid 4-glucoside	45
29	6-Hydroxycoumarin¬†	44
29	3,4-Trihydroxy benzenepropanoic acid	44
29	Dehydroabietic acid	44
32	[(epi)catechin-(epi)gallocatechin]	43
32	Gallic acid	43
34	Pinusolidic acid	42
35	Hydroxypalmitic Acid	41
35	15-Hydroxypinusolidic acid	41
37	Carnosol	40

**Table 3:** Docking details of the selected components of ARBE on HSP90A.

Component	S Score Kcal/mol	H-Bond Interactions	*Pi*-H Interactions
7-oxo-dehydroabietic acid	−6.5439	THR184	ASN51
Caffeic acid	−5.1359	SER52	-
Narcissoside (Isorhamnetin-3-*O*-rutinoside)	−8.4051	ASP102	ASN106
Isorhamnetin-3-*O*-glucoside	−7.6758	ASP93	-
Syringetin-3-*O*-glucoside	−8.0354	ASP93	ASN106
15-hydroxy-7-oxo-dehydroabietic acid	−5.5907	MET98, PHE 138	-
6-*O*-*p*-coumaroyl ajugol	−7.5511	GLU47, ASN 51	-
Luteolin 7-rhamnoside	−7.1269	GLY97	ASN106
Robustaflavone 7,4’-dimethyl ether	−7.0831	MET98	-
Ferulic acid	−5.0937	ASN51	-
T5M	−6.4559	THR184	ASN51

**Table 4:** Docking details of the selected components of ARBE on EGFR.

Component	S Score Kcal/mol	H-Bond Interactions	*Pi*-H Interactions
7-oxo-dehydroabietic acid	−5.7797	MET769, THR830	GLY772
Caffeic acid	−5.2224	MET742, GLN767, ASP831	-
Narcissoside (Isorhamnetin-3-*O*-rutinoside)	−9.0112	MET742, MET769, ASP 831	LEU694
Isorhamnetin-3-*O*-glucoside	−8.42596	MET742, ASP 831	-
Syringetin-3-*O*-glucoside	−8.5239	MET742, ARG817, ASP 831	LEU694
15-hydroxy-7-oxo-dehydroabietic acid	−5.9105	LYS721	VAL702
6-*O*-*p*-coumaroyl ajugol	−7.4587	MET742, GLU780	-
Luteolin 7-rhamnoside	−7.2894	THR766, ASP 831	LEU694, GLY772
Robustaflavone 7,4′-dimethyl ether	−8.4544	LYS721	LEU694, VAL 702
Ferulic acid	−5.1249	MET769	-
erlotinib	−7.76417	VAL702, MET769,	-

**Table 5:** Docking details of the selected components of ARBE on P53.

Component	S Score Kcal/mol	H-Bond Interactions	*Pi*–H Interactions
7-oxo-dehydroabietic acid	−4.9993	GLY262	-
Caffeic acid	−4.5554	SER99	THR256
Narcissoside (Isorhamnetin-3-*O*-rutinoside)	−7.1544	SER96	PRO 98
Isorhamnetin-3-*O*-glucoside	−7.1453	ASP208	THR256, LEU 264
Syringetin-3-*O*-glucoside	−6.7796	SER96, ASP 208, GLY262	PRO98
15-hydroxy-7-oxo-dehydroabietic acid	−5.0645	ASP208	-
6-*O*-*p*-coumaroyl ajugol	−6.7463	GLU 204, ILE 254	-
Luteolin 7-rhamnoside	−5.9100	SER260, ARG 267	-
Robustaflavone 7,4′-dimethyl ether	−6.6223	SER99, ARG 158, ASP 208	THR256
Ferulic acid	−4.5974	SER99, GLY 262	THR256

## Data Availability

All data and materials used are available in the manuscript and supplementary information.

## Supplementary Materials

The following supporting information can be downloaded at: https://www.mdpi.com/article/10.3390/ph15101270/s1, Figure S1: The mass spectra of the identified compounds in ARBE, Table S1: The peak area percentages of the identified metabolites in ARBE (in descending order), Table S2: Pharmacokinetics and the drug-likeness properties of ARBE major compounds, Table S3: Molecular targets of ARBE major compounds, Table S4: Molecular targets associated with RIRI, Table S5: Molecular targets of ARBE associated with RIRI, Table S6: Top 20 ARBE molecular targets associated with RIRI, Table S7: Detailed information of GO analysis for biological processes, Table S8: Detailed information of GO analysis for molecular functions, Table S9: Detailed information of GO analysis for cellular components, Table S10: Detailed information of KEGG pathway analysis, Table S11: Crystal structures of HSP90A, EGFR, and P53
