# Supplementary material for: Agathis robusta Bark Extract Protects from Renal Ischemia-Reperfusion Injury: Phytochemical, In Silico and In Vivo Studies
Source: Pharmaceuticals (Basel). 2022 Oct 14;15(10):1270. doi: 10.3390/ph15101270 (PMC9610891; doi:10.3390/ph15101270)

Figure S1: The mass spectra of the identified metabolites in *Agathis robusta* bark extract by LC-ESI-MS/MS (negative and positive mode ESI).

Quinic acid (1)

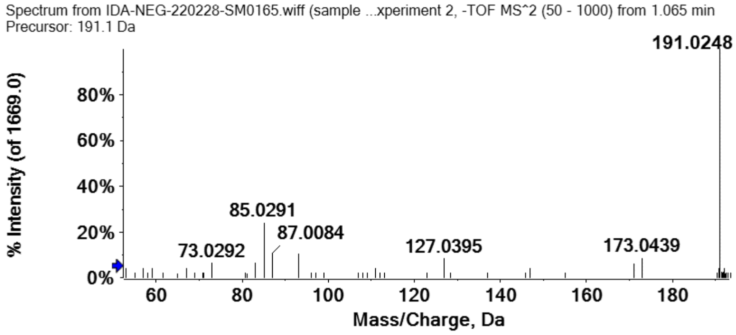

Trihydroxy benzoic acid (gallic acid) (2)

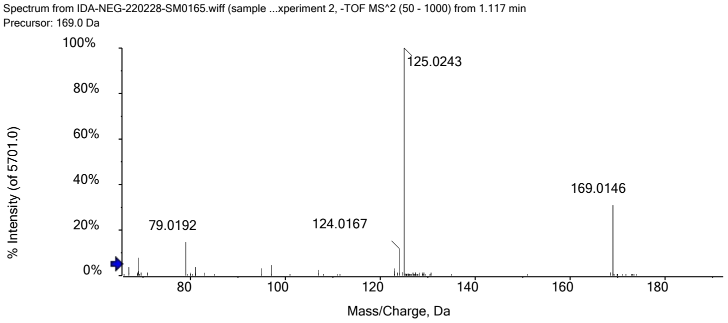

Succinic acid (3)

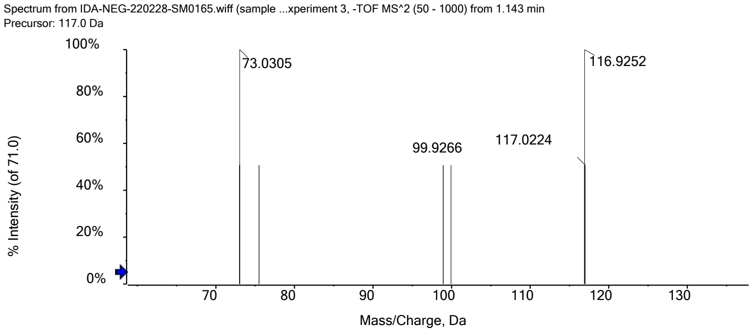

Dihydroxybenzoic acid (protocatechuic acid) (4)

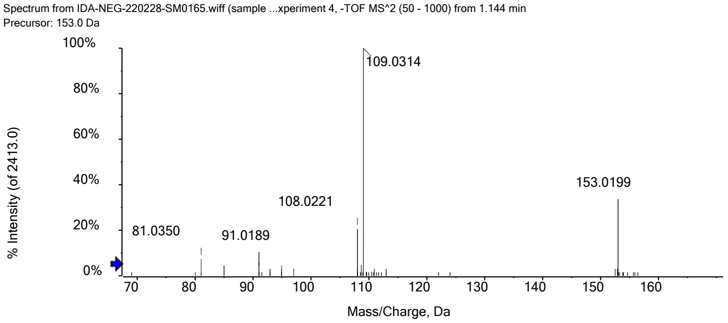

Dihydrocaffeic acid [3-(3,4-Dihydroxy-phenyl)propionic acid] (5)

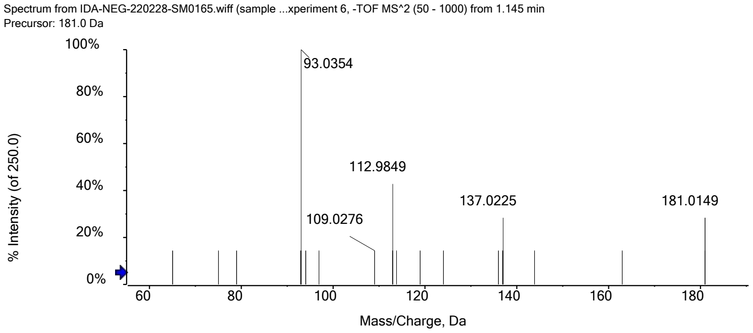

β,3,4-Trihydroxy benzenepropanoic acid* (6)

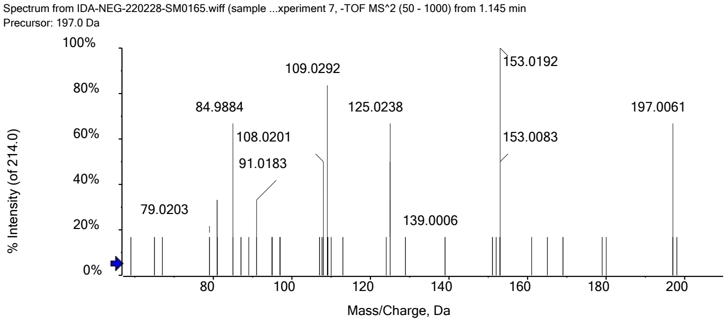

N-methyl alanine (7)

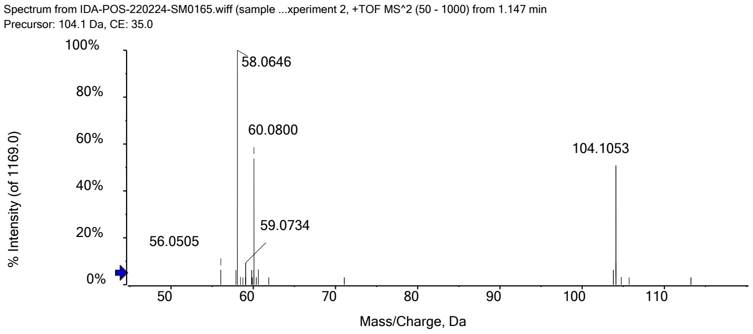

1,2-Benzenediol (8)

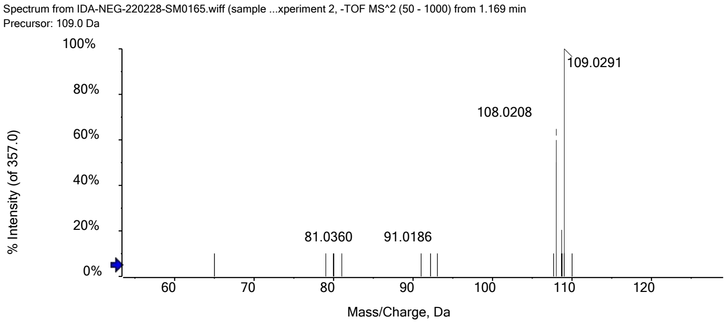

Choline (9)

Spectrum from IDA-POS-220224-SM0165.wiff (sample ...xperiment 2, +TOF MS^2 (50 - 1000) from 1.173 min
Precursor: 104.1 Da, CE: 35.0

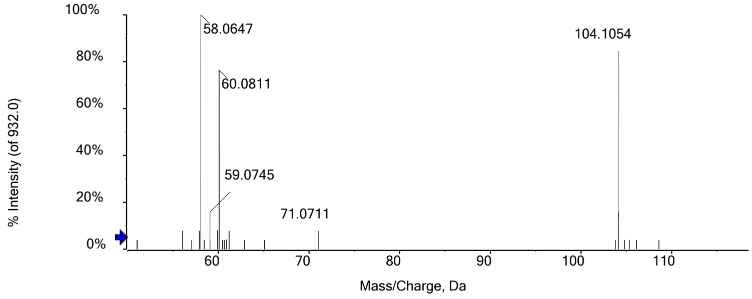

Salicylic acid (10)

Spectrum from IDA-NEG-220228-SM0165.wiff (sample ...xperiment 3, -TOF MS^2 (50 - 1000) from 1.182 min
Precursor: 137.0 Da

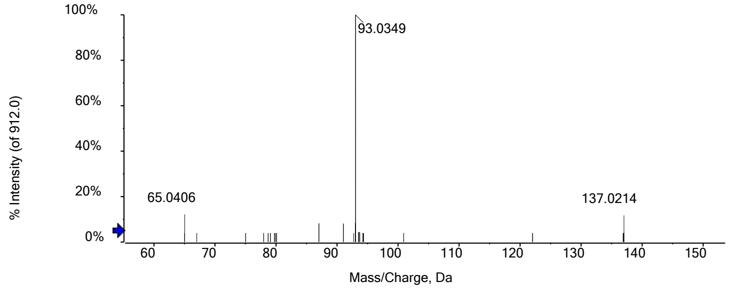

Sorbitol 6-phosphate (11)

Spectrum from IDA-NEG-220228-SM0165.wiff (sample ...xperiment 9, -TOF MS^2 (50 - 1000) from 1.185 min
Precursor: 261.0 Da

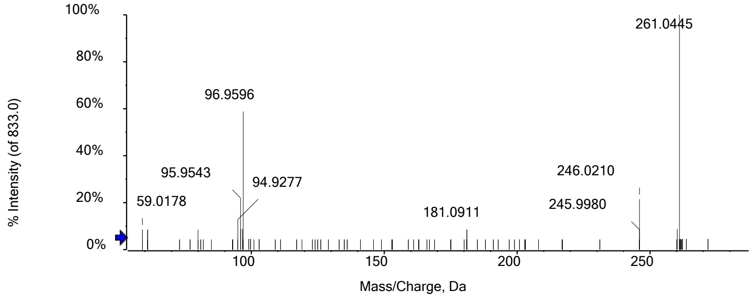

4- Aminophenol (12)

Spectrum from IDA-POS-220224-SM0165.wiff (sample ...xperiment 2, +TOF MS^2 (50 - 1000) from 1.186 min
Precursor: 110.1 Da, CE: 35.0

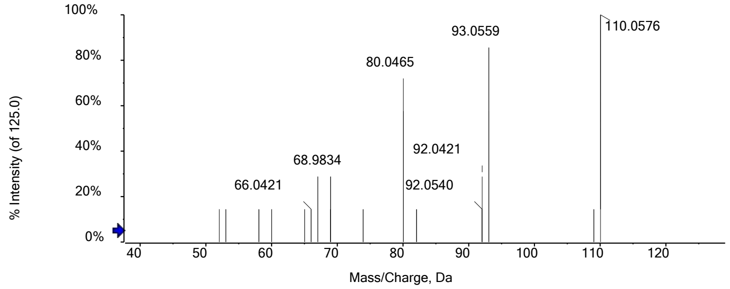

Adenine (13)

Spectrum from IDA-POS-220224-SM0165.wiff (sample ...xperiment 2, +TOF MS^2 (50 - 1000) from 1.211 min
Precursor: 136.1 Da, CE: 35.0

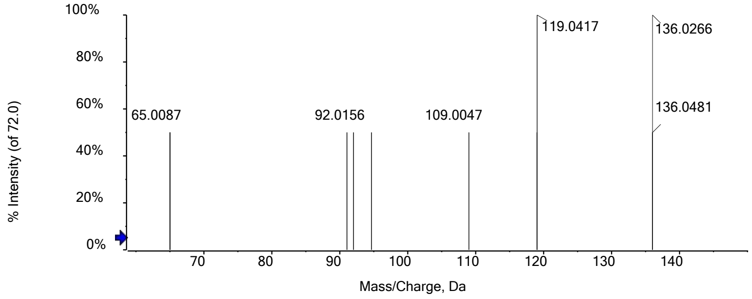

Protocatechuic acid 4- glucoside (14)

Spectrum from IDA-NEG-220228-SM0165.wiff (sample ...periment 12, -TOF MS^2 (50 - 1000) from 1.237 min
Precursor: 315.1 Da

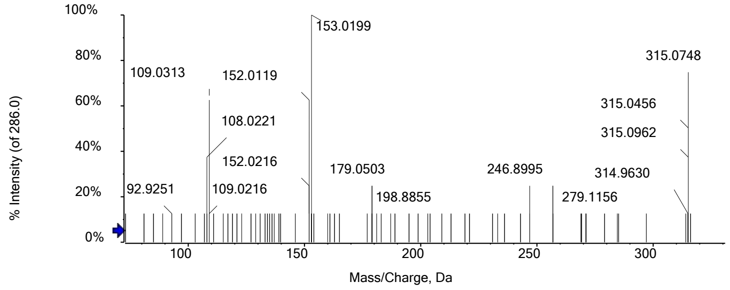

Shikimic acid (15)

Spectrum from IDA-NEG-220228-SM0165.wiff (sample ...xperiment 2, -TOF MS^2 (50 - 1000) from 1.246 min
Precursor: 173.1 Da

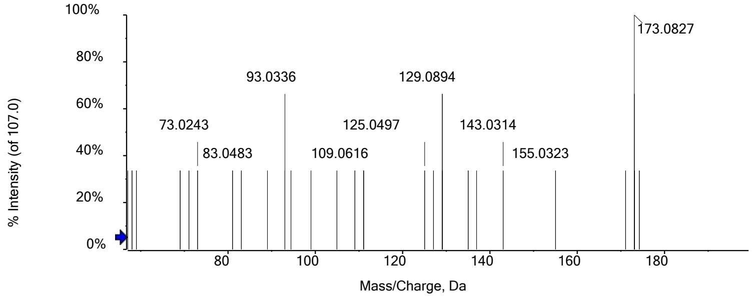

Methoxy hydroxy benzoic acid (Vanillic acid) (16)

Spectrum from IDA-NEG-220228-SM0165.wiff (sample ...xperiment 2, -TOF MS^2 (50 - 1000) from 1.349 min
Precursor: 167.0 Da

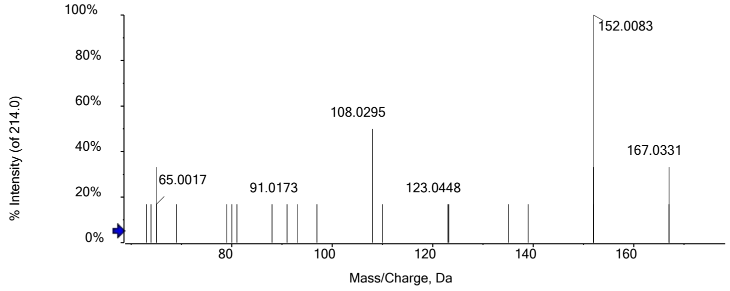

N,*N*,*N*-trimethyl-l-alanyl-l-proline betaine (17)

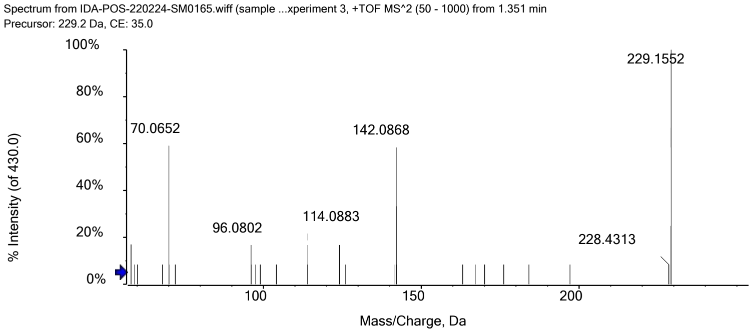

Caffeic acid (18)

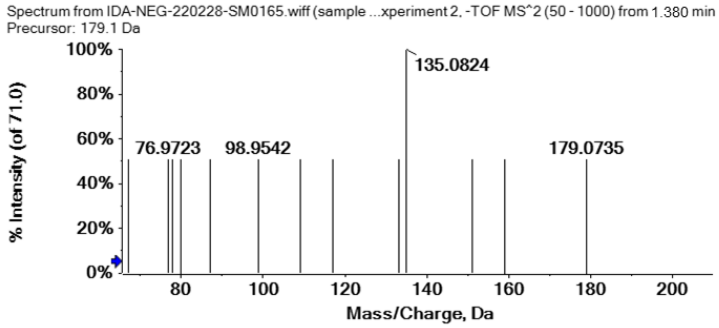

Ferulic acid (19)

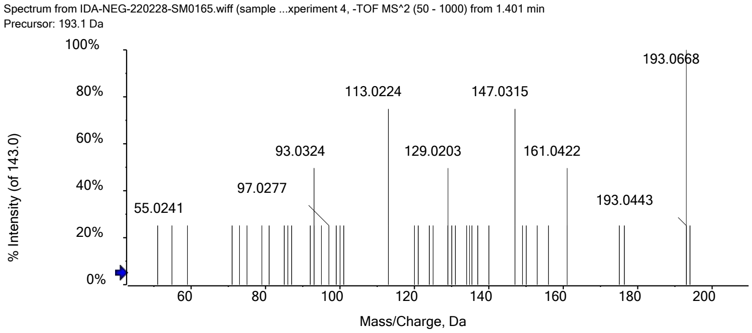

Dihydrosinapic acid (20)

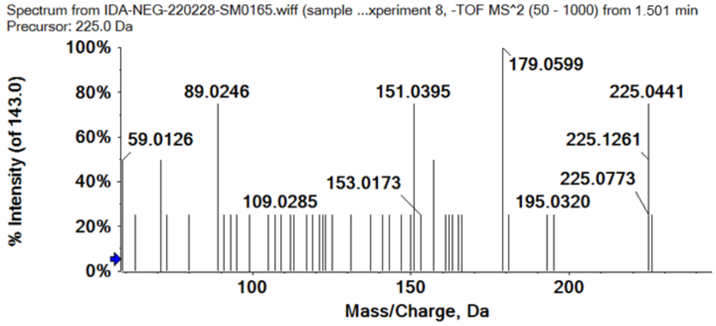

6-Hydroxycoumarin (21)

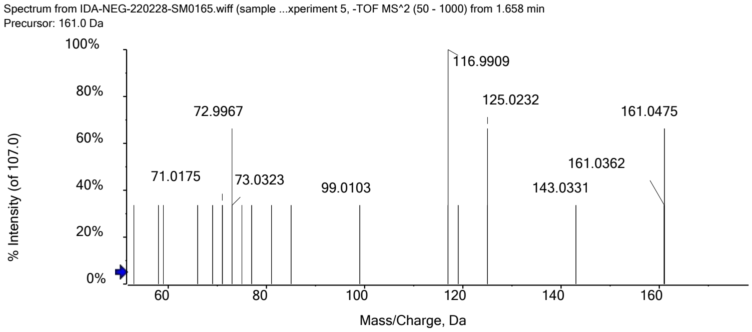

Glycine- betaine (22)

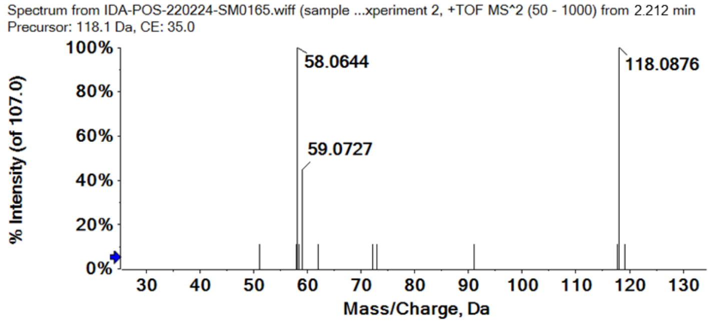

[(epi)gallocatechin-(epi)gallocate-chin] (23)

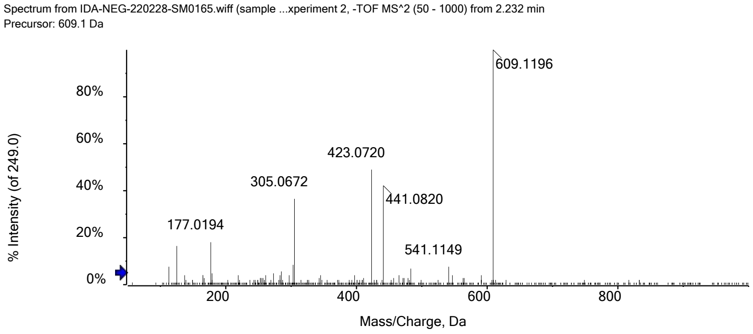

[(epi)catechin-(epi)gallocatechin] (24)

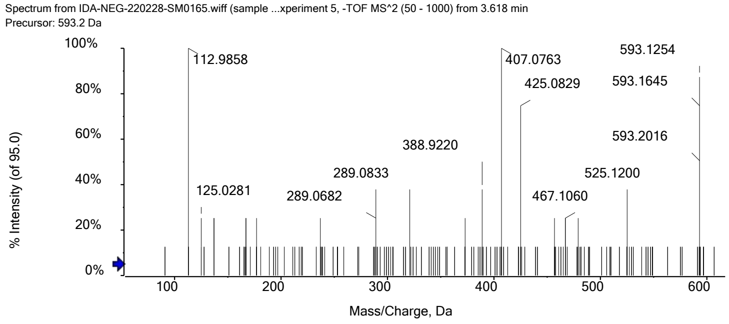

[(epi)catechin-(epi)gallocatechin] (25)

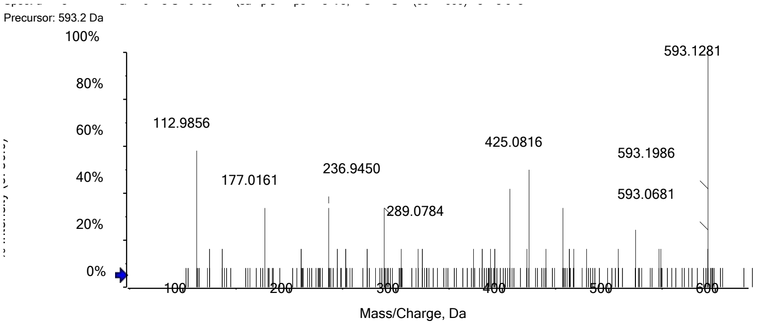

[(epi)catechin-(epi)gallocatechin] (26)

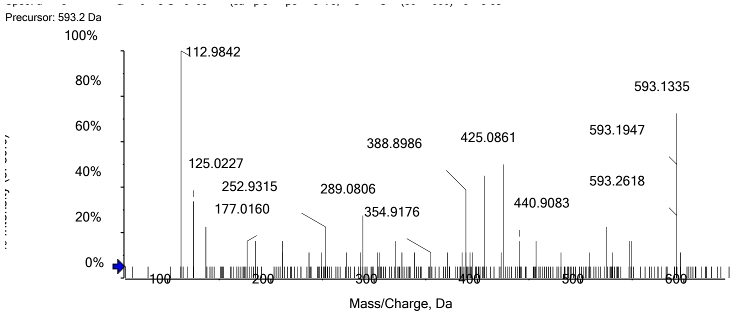

[(epi)gallocatechin-(epi)gallocatechin] (27)

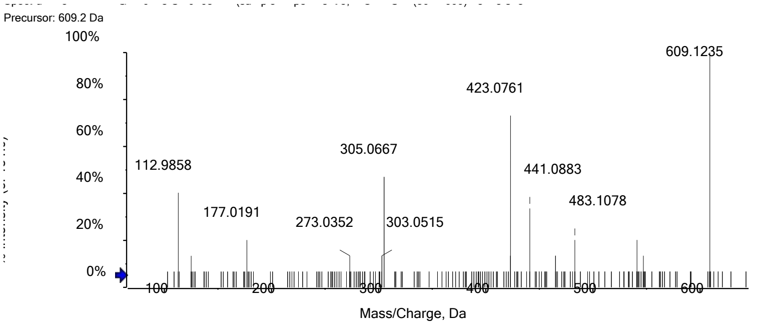

(epi)gallocatechin (28)

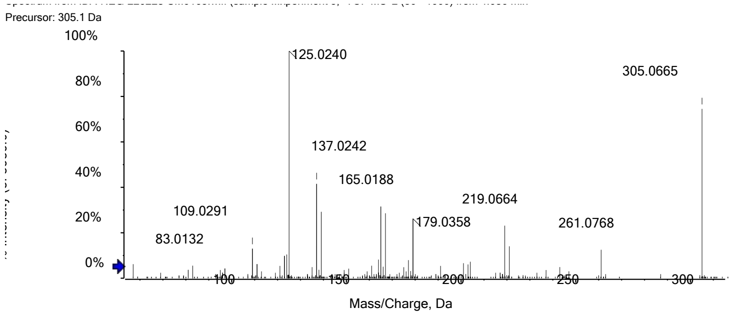

[(epi)gallocatechin-(epi)gallocatechin]

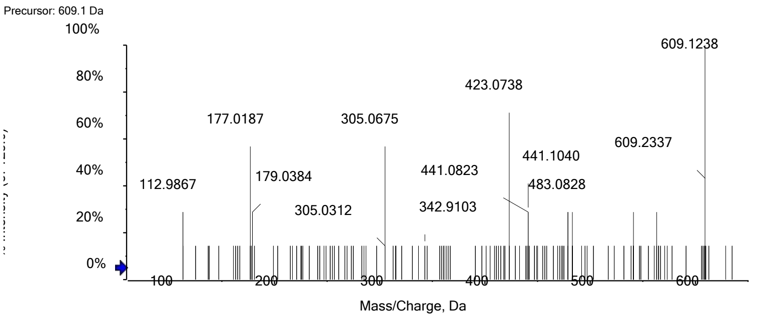

Cysteine (30)

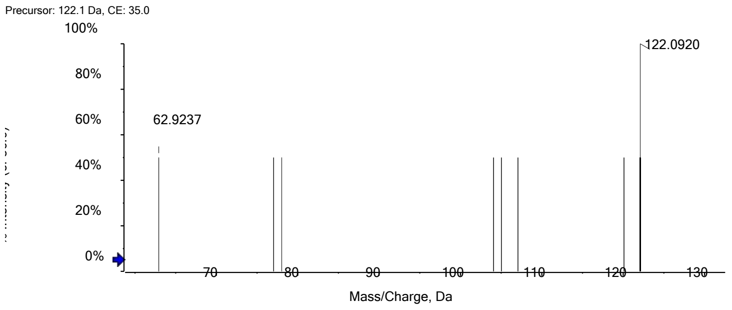

Hydroxycaffeic acid* (31)

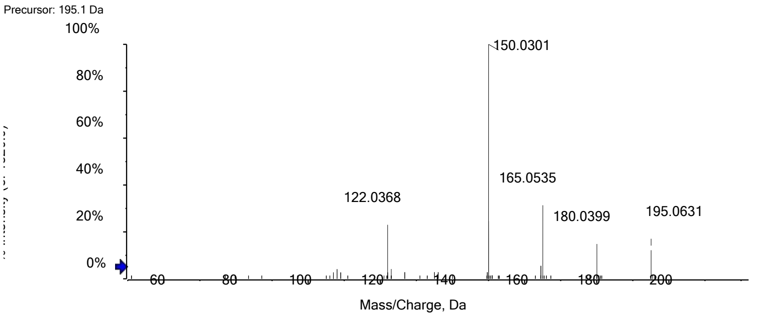

Isorhamnetin-*O*-glucoside (32)

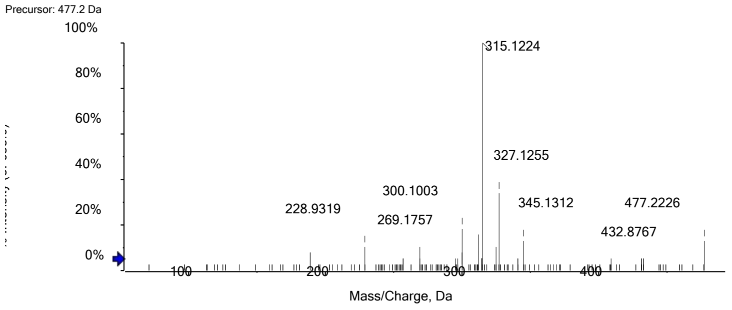

Hypoxanthine (33)

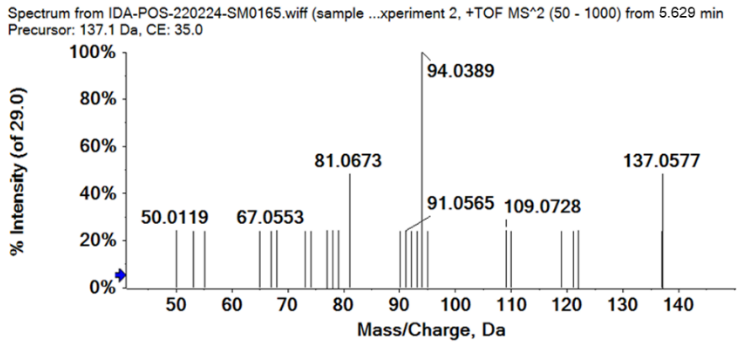

3,4,3',5'-Tetrahydroxystilbene 3'-glucoside (34)

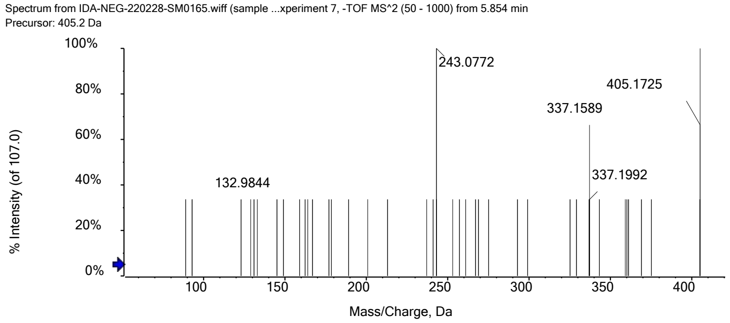

6,7-dihydroxycoumarin (esculetin) (35)

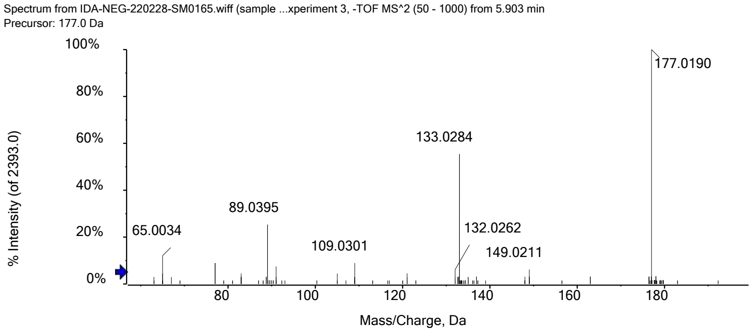

Quercetin-rhamnose-hexose (36)

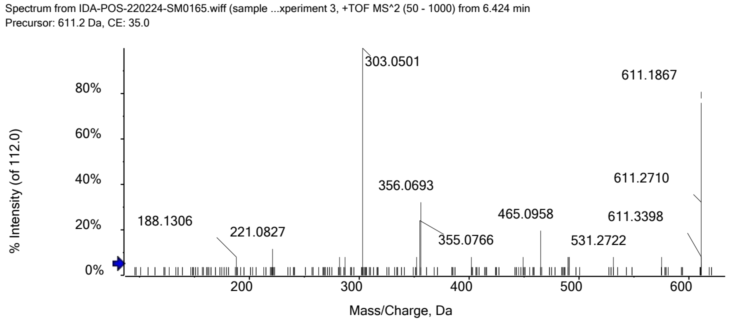

Formononetin (37)

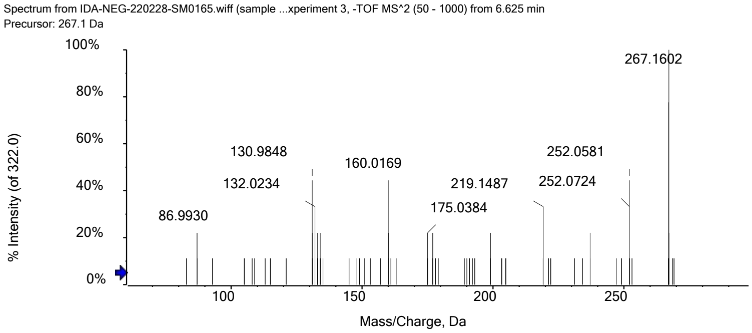

Danshensu (*α*,3,4-trihydroxy benzenepropanoic acid) (38)

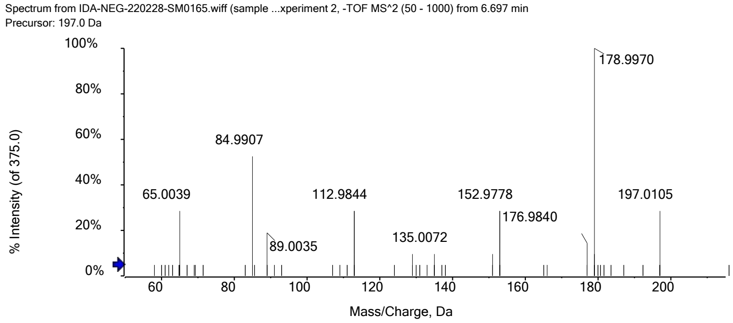

Kaempferol-7-neohesperidoside (39)

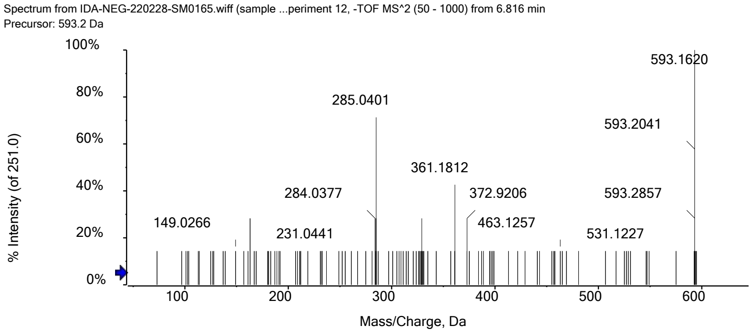

Quercetin hexoside (40)

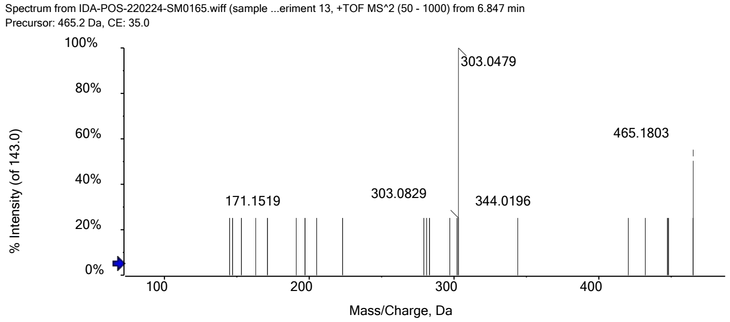

Okanin-4'-*O*-glucoside [2',3',4',3,4-pen-tahydroxychalcone glucoside] (41)

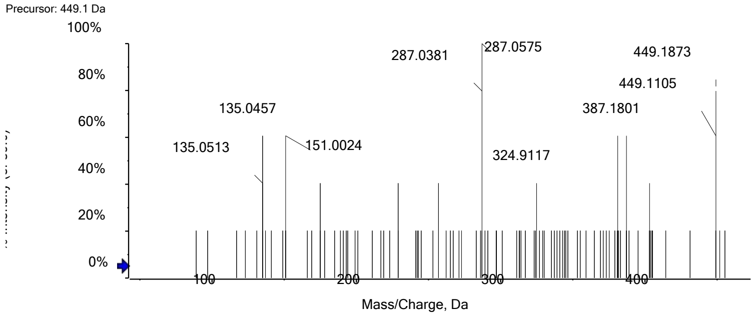

Isorhamnetin-3-*O*-rutinoside (narcissoside) (42)

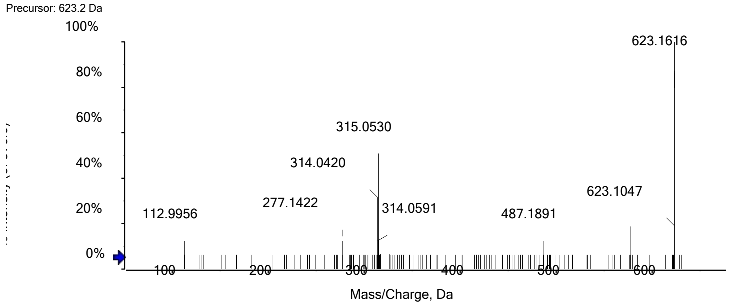

6-*O*-*p*-coumaroyl ajugol (43)

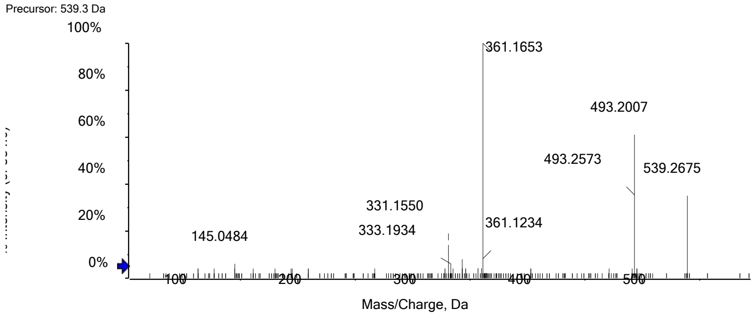

Agatharesinol (44)

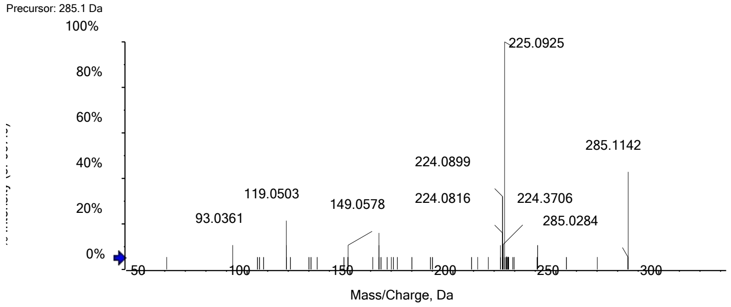

Isorhamnetin-3-*O*-glucoside (45)

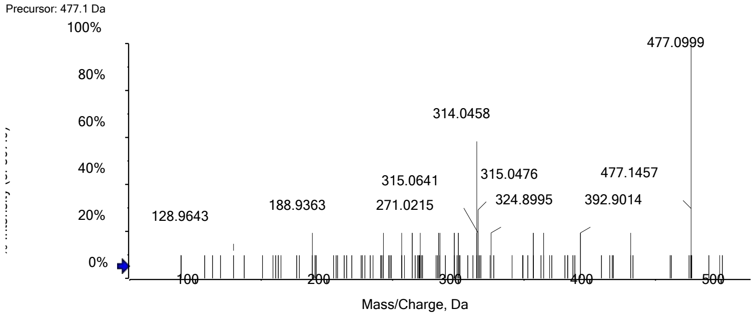

(epi)catechin (46)

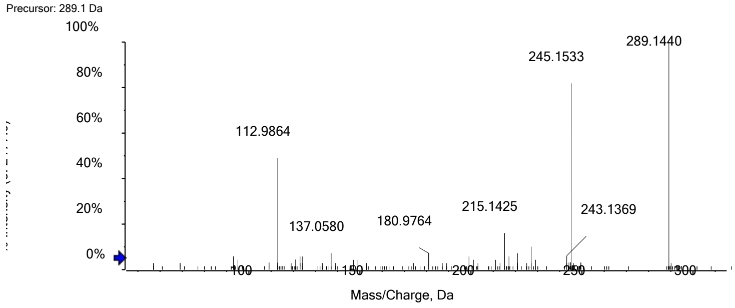

Kaempferol hexoside (47)

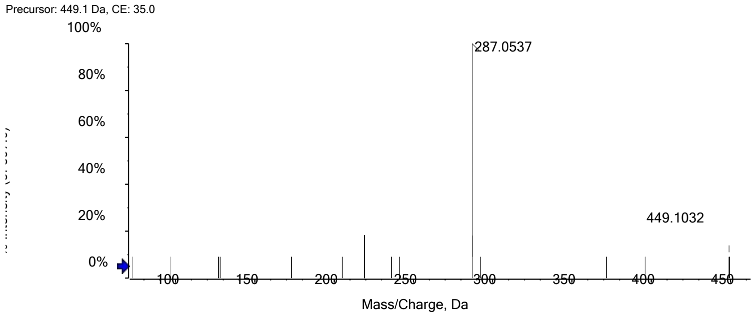

3, 5, 7-trihydroxy-4'-methoxyflavone (Diosmetin) (48)

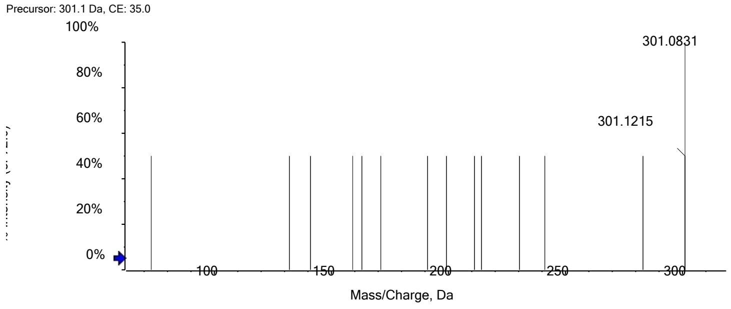

Apigenin-*O*-hexoside (49)

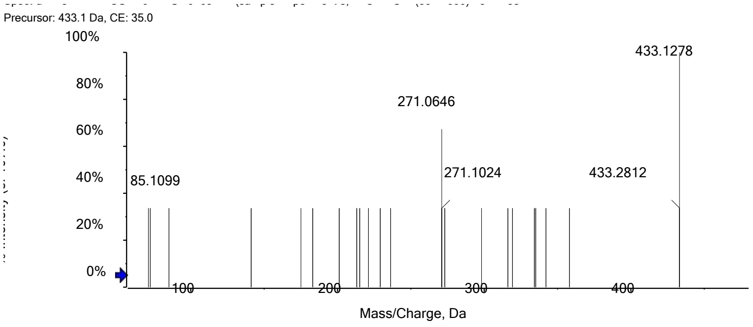

Phlorizin (50)

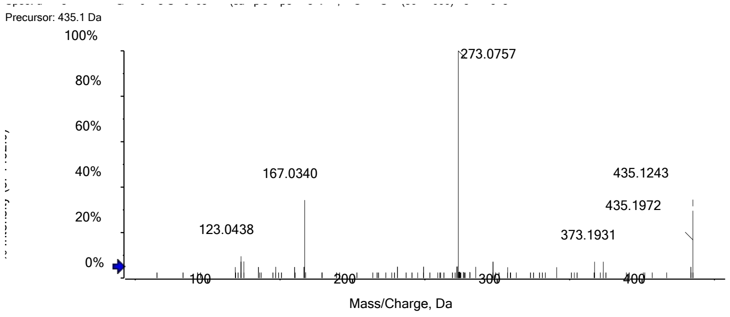

Syringetin-3-*O*-glucoside (51)

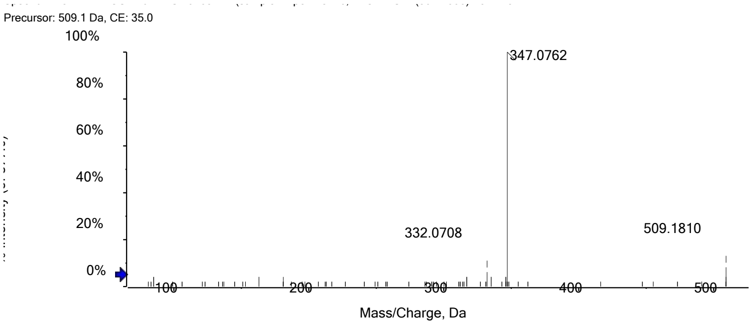

7-Hydroxy-8-methoxydedihydro-rutaecarpine (7-Hydroxy-8-methoxy,7,8-Dehydro-rutaecarpine) (52)

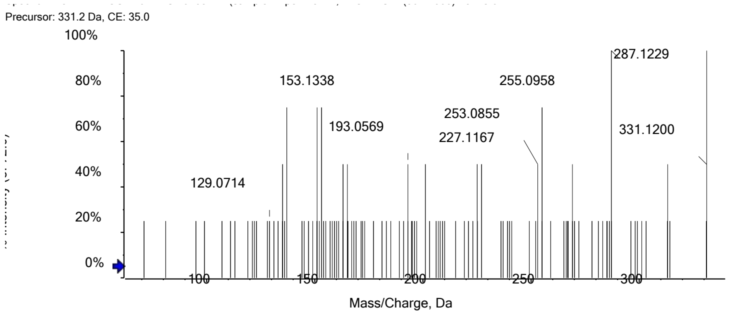

Myricetin (53)

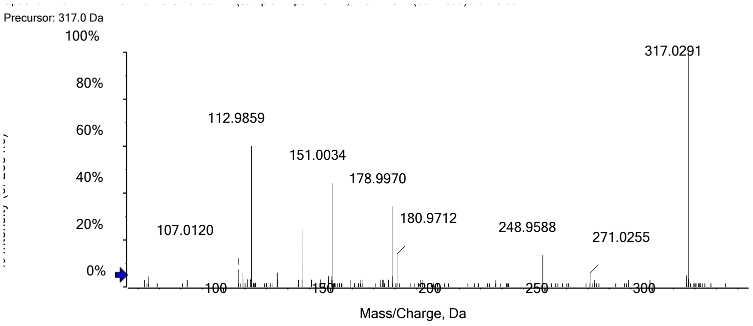

Umbelliferone (54)

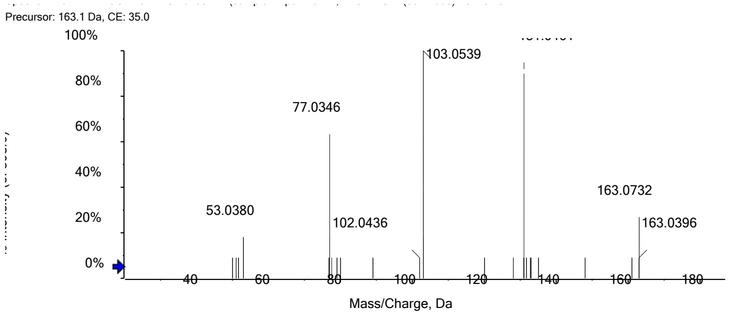

Hymecromone (4-methylumbellifer-one) (55)

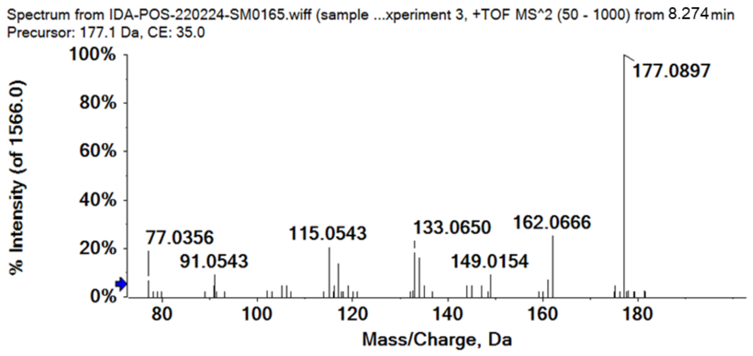

luteolin 7-*O*-rhamnoside (56)

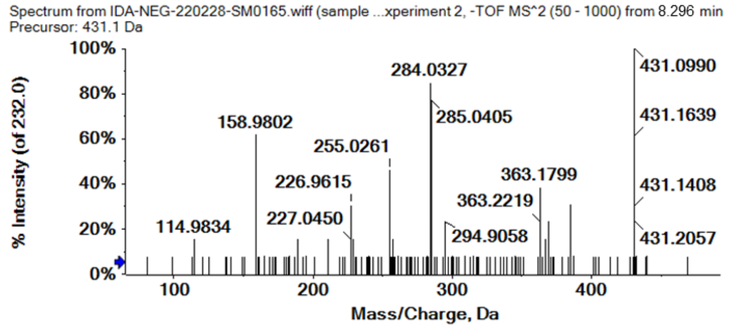

Steviol (57)

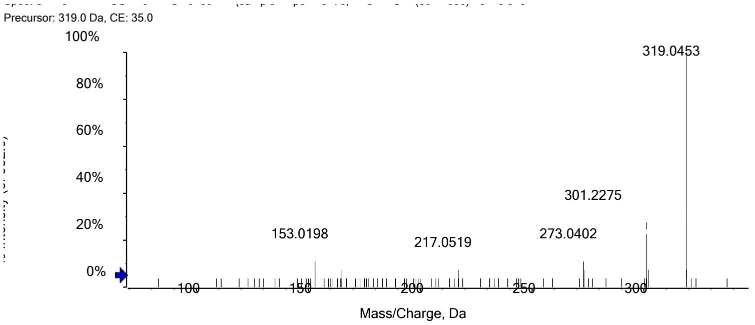

16-Hydroxy-8(17),13-labdadien-15,16-olid-19-oic acid (58)

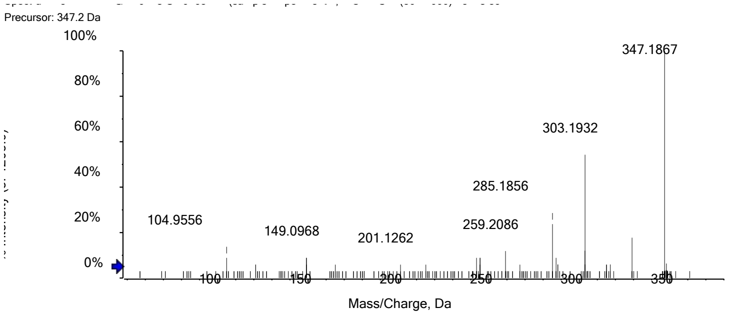

Angelol A (59)

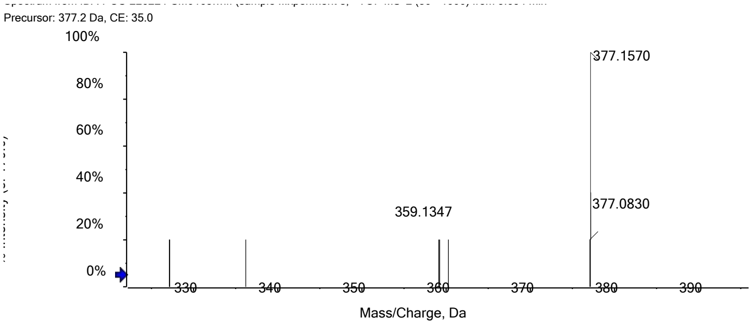

6-*O*-*p*-coumaroyl dihydroajugol (60)

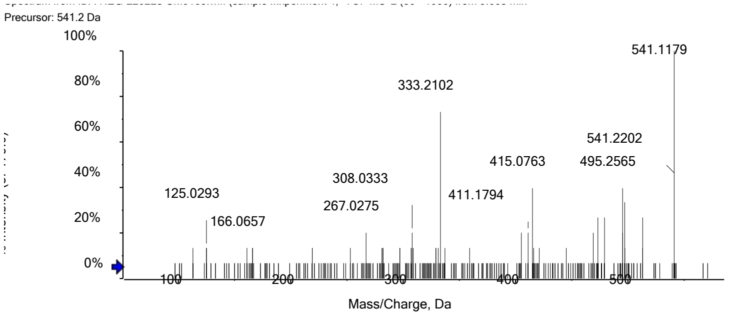

Copalic acid (61)

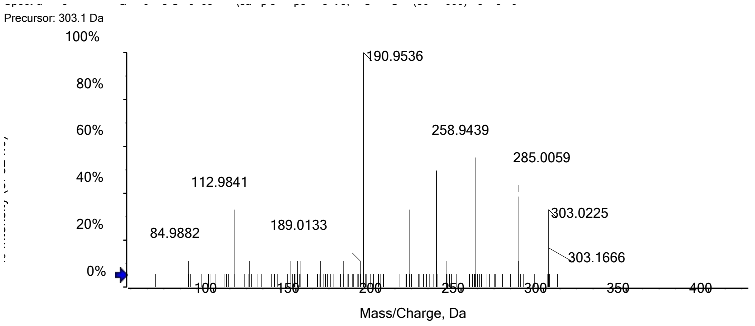

Abietic acid (62)

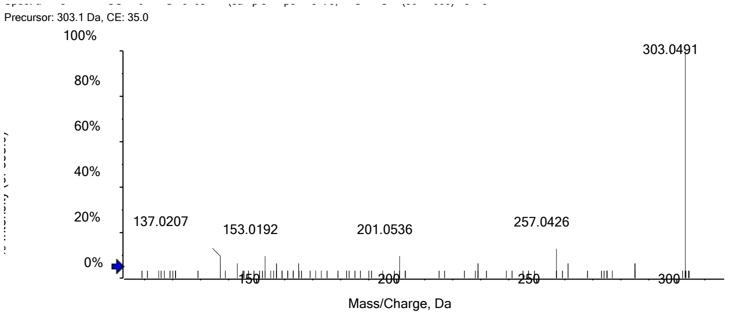

Retinoic acid (63)

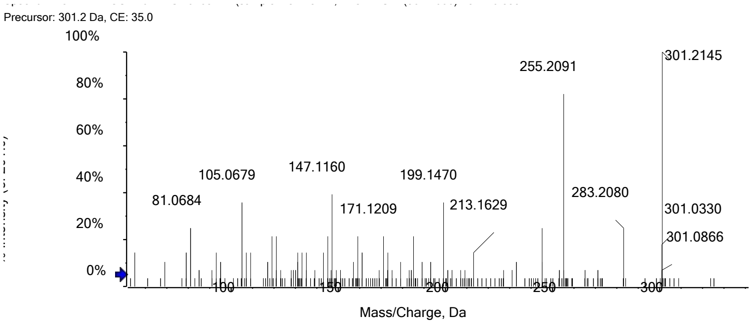

Dehydroabietic acid (64)

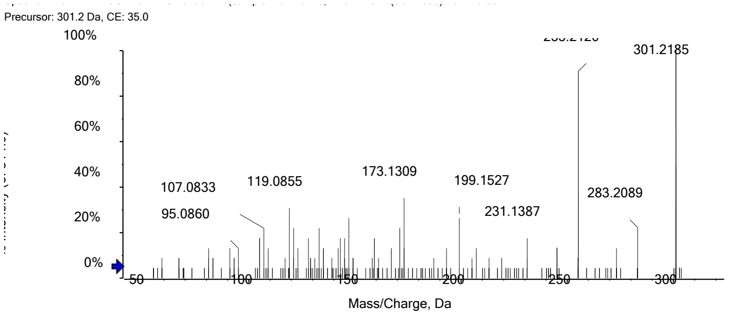

Taxifolin (dihydroquercetin) (65)

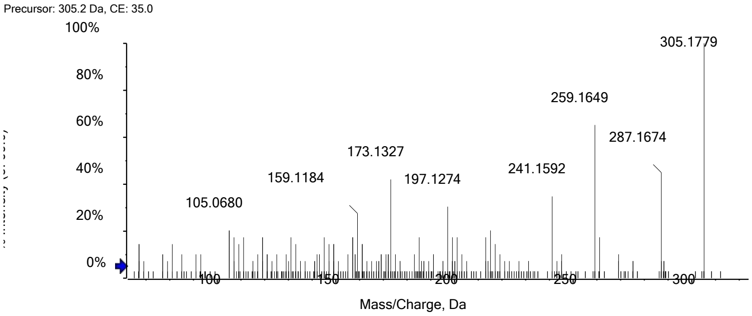

Luteolin (66)

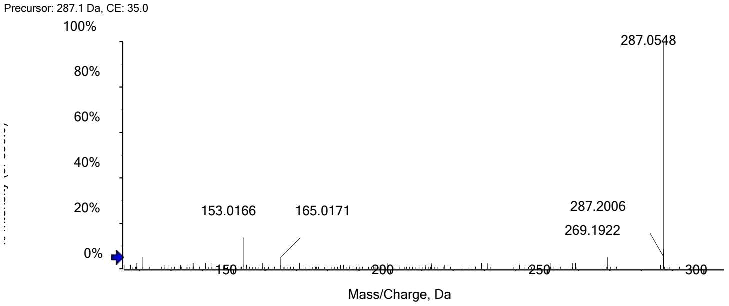

15-Hydroxypinusolidic acid (67)

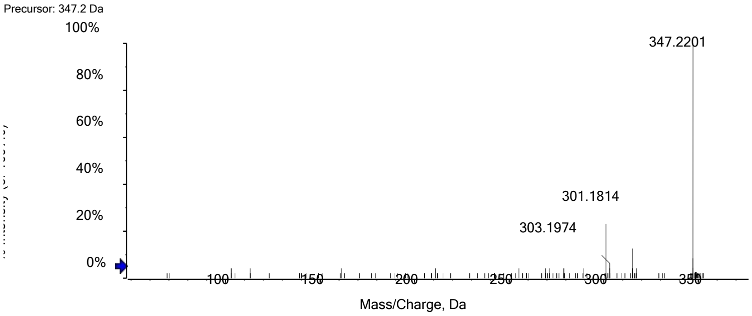

Isoangelol (68)

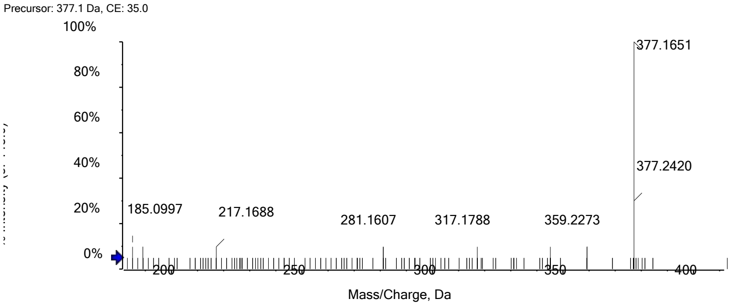

(epi)gallocatechin-(epi)gallocatechin-(epi)catechin (69)

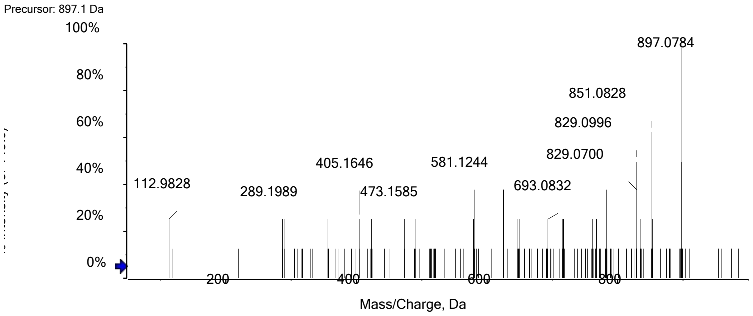

(epi)catechin-(epi)catechin (70)

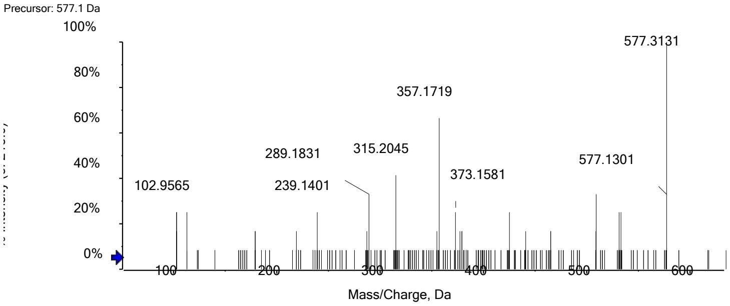

3 -hydroxy-(13S)-l6-nor-pimar-7-en-l5-oic acid* (71)

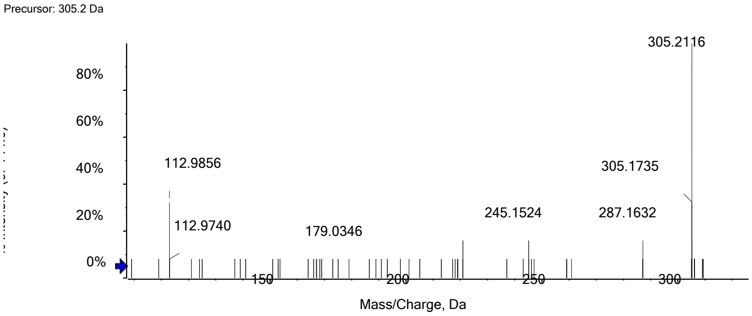

15-hydroxy-7-oxo-dehydroabietic acid (72)

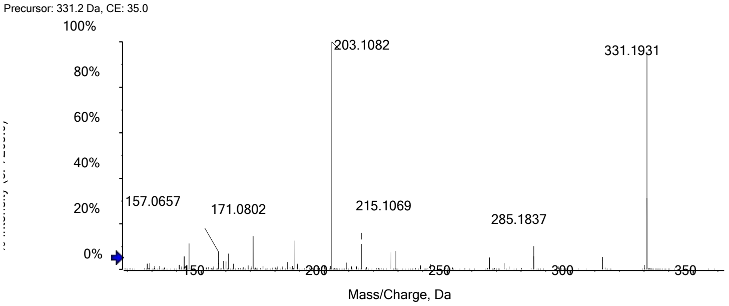

Carnosol (73)

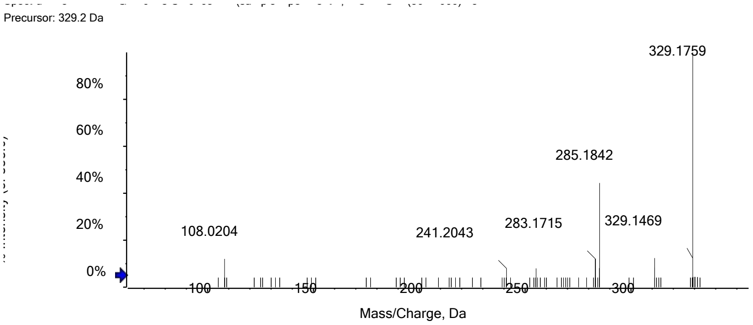

Quercetin (74)

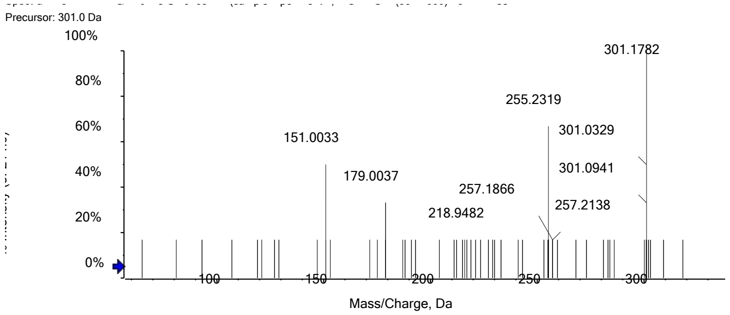

Isoginkgetin (75)

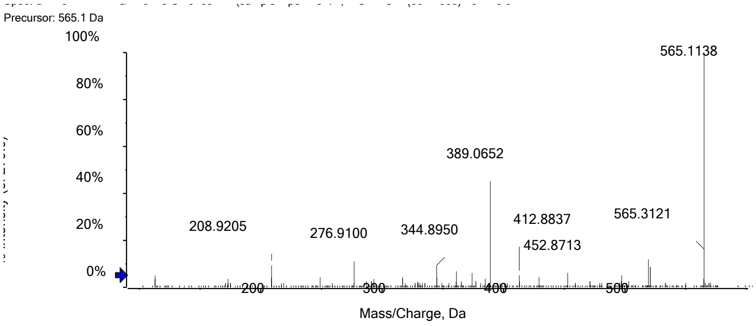

7-oxo-dehydroabietic acid (76)

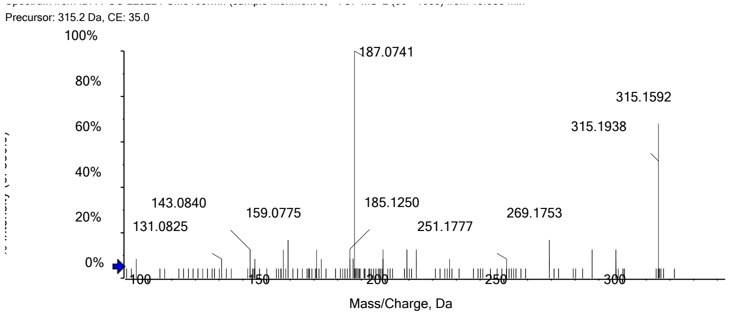

Chrysin-6-methyl-ether (77)

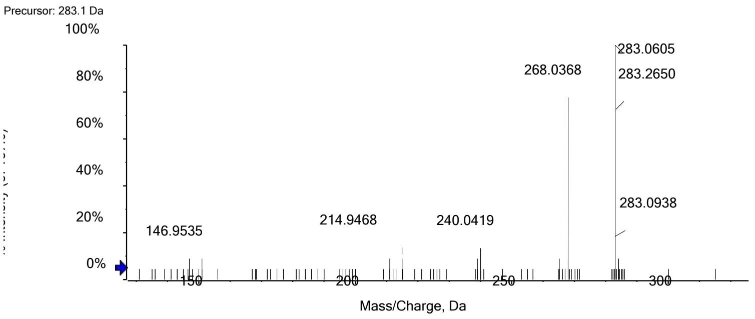

Physcion (78)

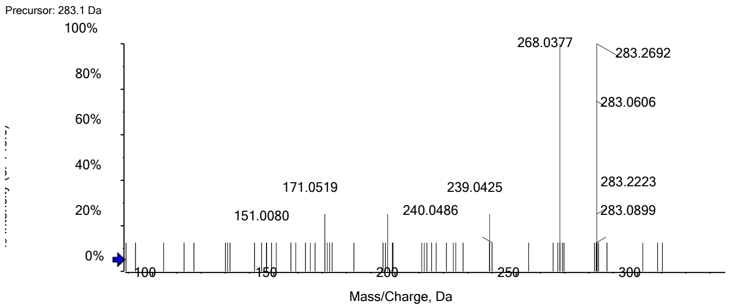

Pinusolidic acid* (79)

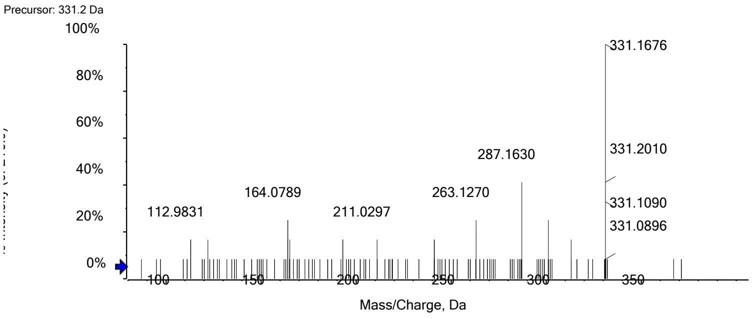

Hydroxypalmitic Acid (80)

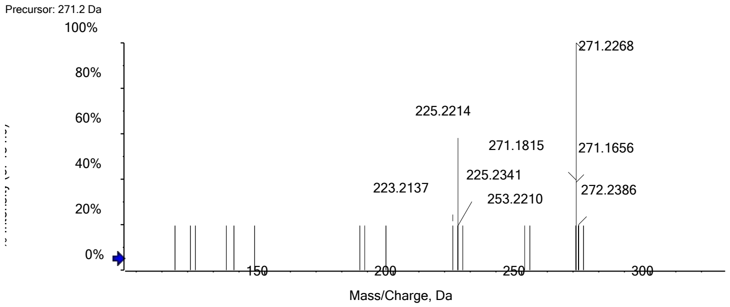

4'-Hydroxywogonin (81)

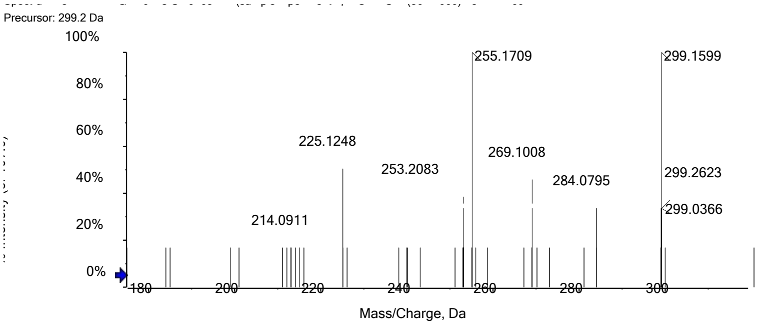

2'',3''-Dihydro-3',3'''-biapigenin (82)

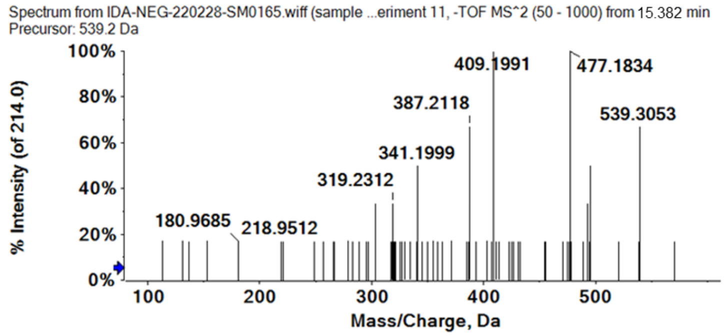

2',8''-Biapigenin (83)

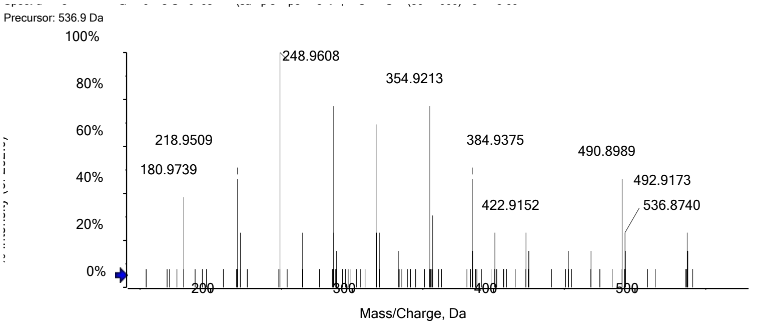

Robustaflavone 7,4'-dimethyl ether (84)

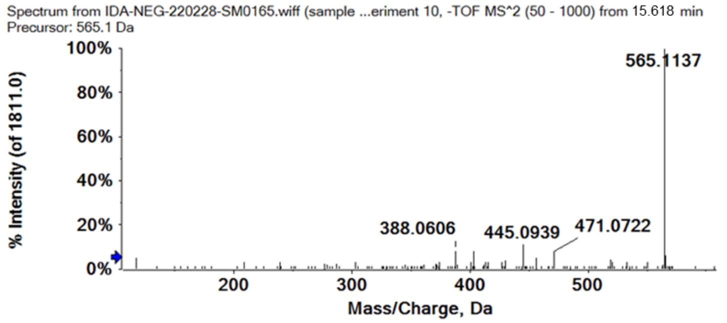

Delicaflavone (85)

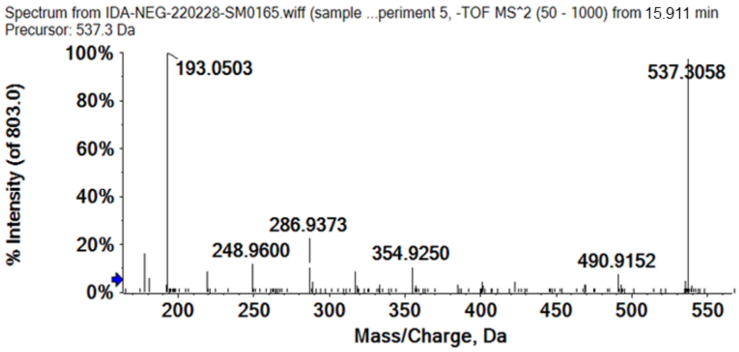

Abietic acid isomer (86)

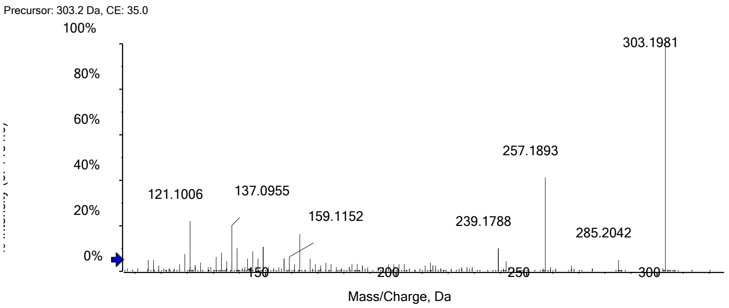

Dehydroabietic acid isomer (87)

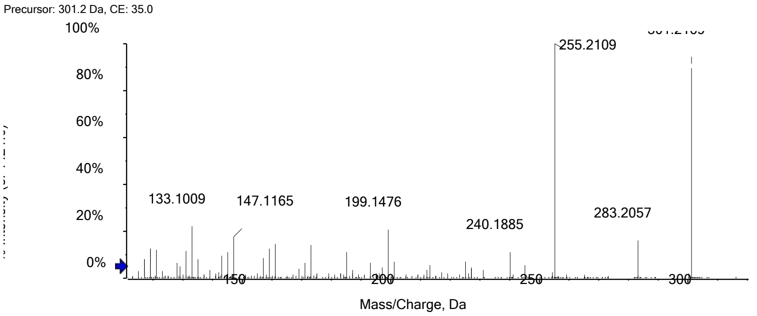

Dehydrated 15-hydroxy-7-oxo-dehy-droabietic acid (88)

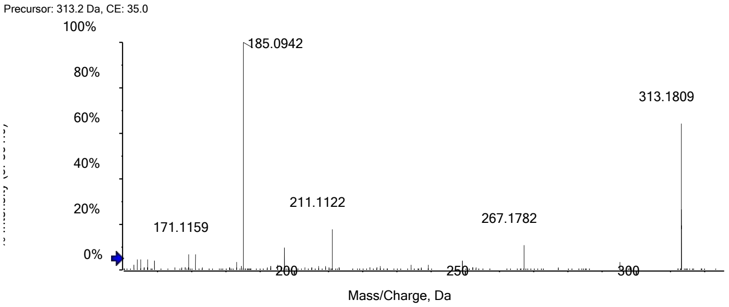

Agarotetrol formate adduct (89)

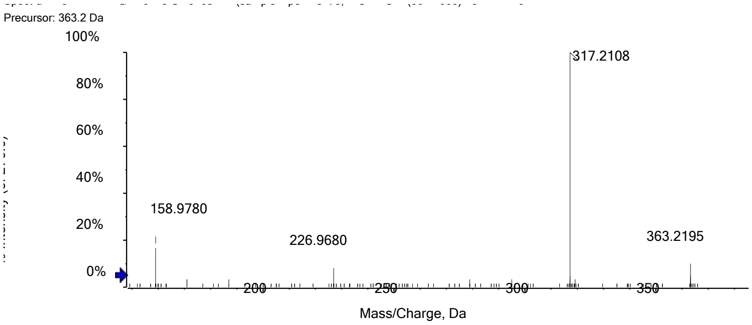

7-oxo-dehydroabietic acid isomer (90)

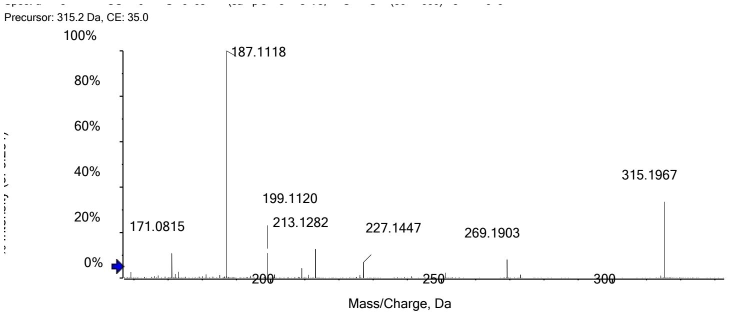

Abietic acid isomer (91)

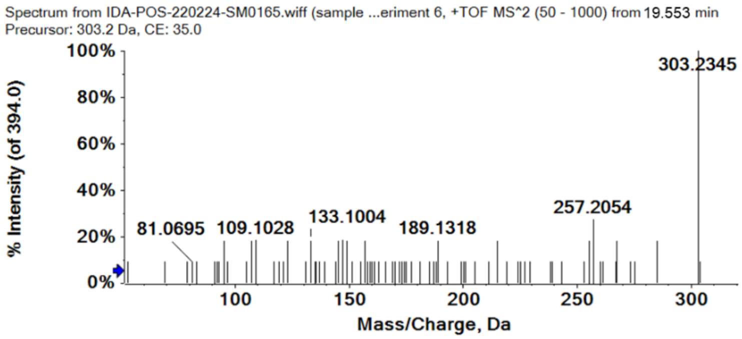

Dehydroabietic acid isomer (92)

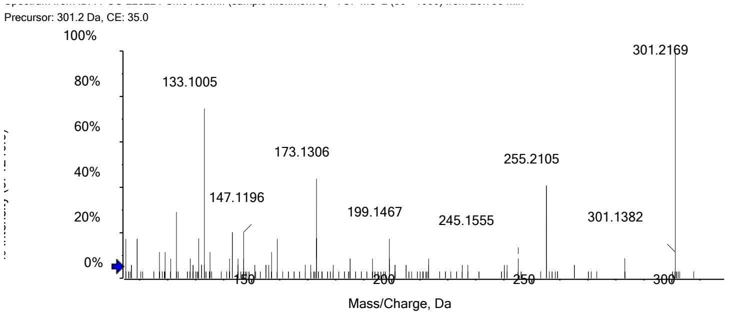

Dehydroabietic acid isomer (93)

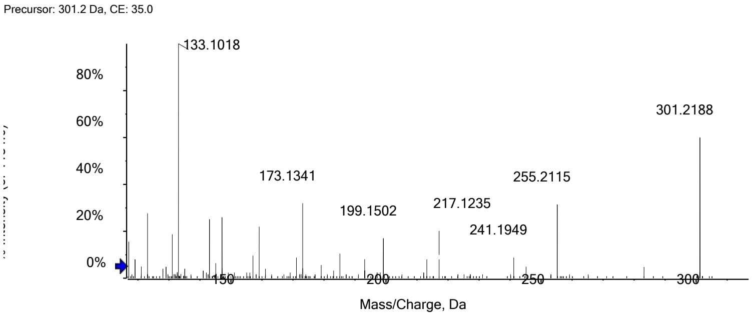

Dehydroabietic acid isomer (94)

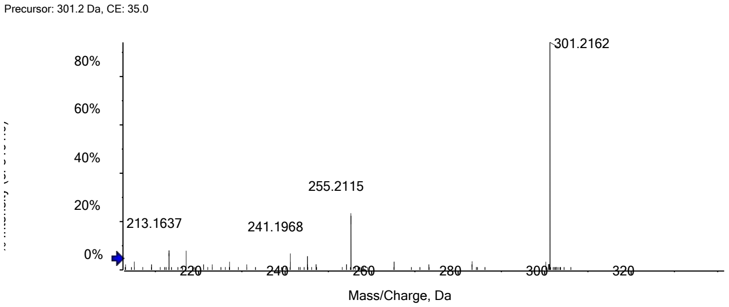

7-oxo-dehydroabietic acid isomer (95)

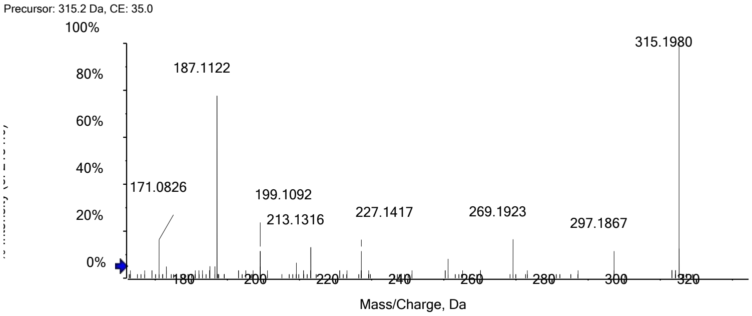

Supplement: Supplementary file 1 [file pharmaceuticals-15-01270-s001.zip › Figure S1.pdf]
