# Supplementary material for: Agathis robusta Bark Extract Protects from Renal Ischemia-Reperfusion Injury: Phytochemical, In Silico and In Vivo Studies
Source: Pharmaceuticals (Basel). 2022 Oct 14;15(10):1270. doi: 10.3390/ph15101270 (PMC9610891; doi:10.3390/ph15101270)
Supplement: Supplementary file 1 [file pharmaceuticals-15-01270-s001.zip › Table S11.pdf]

Table S10: The target proteins used for docking of the selected components of *A. robusta* bark extract

Proteins (PDB)	Resolution	co-crystallized ligand	Structure of the co-crystallized ligand	RMSD	Energy score (S) (kcal/mol)
Structure of HSP90 with small molecule inhibitor bound (2XHX)	2.80 Å	2-Tert-butyl-4-(1,3-dihydro-2h-isoindol-2-ylcarbonyl)phenol (T5M)	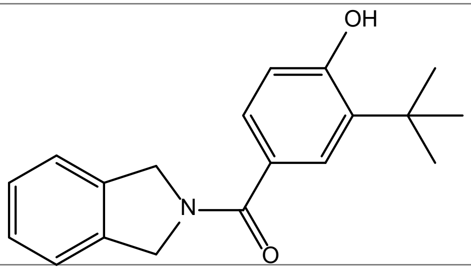	1.2702	- 6.4559
Growth Factor Epidermal Receptor tyrosine kinase domain with 4-anilinoquinazoline inhibitor erlotinib (1M17)	2.60 Å	erlotinib (AQ4)	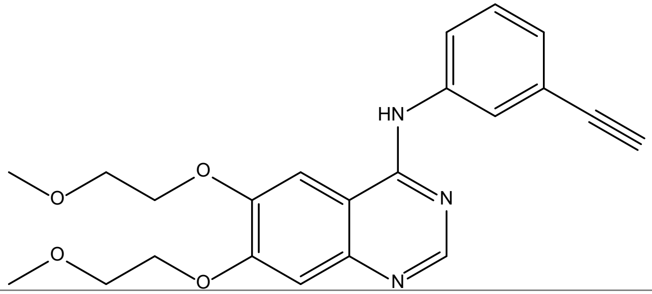	1.1915	-8.1587
An induced fit mechanism regulates p53 DNA binding kinetics to confer sequence specificity (3Q01)	2.10 Å	-	-	-	-
